# The role of co-opted ESCRT proteins and lipid factors in protection of tombusviral double-stranded RNA replication intermediate against reconstituted RNAi in yeast

**DOI:** 10.1371/journal.ppat.1006520

**Published:** 2017-07-31

**Authors:** Nikolay Kovalev, Jun-ichi Inaba, Zhenghe Li, Peter D. Nagy

**Affiliations:** 1 Department of Plant Pathology, University of Kentucky, Lexington, Kentucky, United States of America; 2 Institute of Biotechnology, State Key Laboratory of Rice Biology, Zhejiang University, Hangzhou, P. R. China; University of California Riverside, UNITED STATES

## Abstract

Reconstituted antiviral defense pathway in surrogate host yeast is used as an intracellular probe to further our understanding of virus-host interactions and the role of co-opted host factors in formation of membrane-bound viral replicase complexes in protection of the viral RNA against ribonucleases. The inhibitory effect of the RNA interference (RNAi) machinery of *S*. *castellii*, which only consists of the two-component *DCR1* and *AGO1* genes, was measured against tomato bushy stunt virus (TBSV) in wild type and mutant yeasts. We show that deletion of the co-opted ESCRT-I (endosomal sorting complexes required for transport I) or ESCRT-III factors makes TBSV replication more sensitive to the RNAi machinery in yeast. Moreover, the lack of these pro-viral cellular factors in cell-free extracts (CFEs) used for *in vitro* assembly of the TBSV replicase results in destruction of dsRNA replication intermediate by a ribonuclease at the 60 min time point when the CFE from wt yeast has provided protection for dsRNA. In addition, we demonstrate that co-opted oxysterol-binding proteins and membrane contact sites, which are involved in enrichment of sterols within the tombusvirus replication compartment, are required for protection of viral dsRNA. We also show that phosphatidylethanolamine level influences the formation of RNAi-resistant replication compartment. In the absence of peroxisomes in *pex3Δ* yeast, TBSV subverts the ER membranes, which provide as good protection for TBSV dsRNA against RNAi or ribonucleases as the peroxisomal membranes in wt yeast. Altogether, these results demonstrate that co-opted protein factors and usurped lipids are exploited by tombusviruses to build protective subcellular environment against the RNAi machinery and possibly other cellular ribonucleases.

## Author summary

Positive-strand RNA viruses build membranous replication compartment to support their replication in the infected hosts. One of the proposed functions of the usurped subcellular membranes is to protect the viral RNA from recognition and destruction by various cellular RNA sensors and ribonucleases. To answer this fundamental question on the putative role of co-opted host factors and membranes in protecting the viral double-stranded RNA replication intermediate during replication, the authors took advantage of yeast (*Saccharomyces cerevisiae*), which lacks the conserved RNAi machinery, as a surrogate host for TBSV. The reconstituted RNAi machinery from *S*. *castellii* in *S*. *cerevisiae* was used as an intracellular probe to study the effect of various co-opted cellular proteins and lipids on the formation of RNAi-insensitive replication compartment. Overall, the authors demonstrate the interaction between the RNAi machinery and the viral replicase complex, and the essential roles of usurped host factors in protecting the viral dsRNA replication intermediate from RNAi-based degradation.

## Introduction

One of the hallmark features of positive-strand (+)RNA viruses, including tomato bushy stunt virus (TBSV), is to assemble numerous membrane-bound viral replicase complexes (VRCs) that leads to replication of the viral genomic RNA inside the infected cells. These viruses co-opt subcellular membranes and alter lipid metabolism in addition to usurping host proteins to form replication compartment or organelle. For several viruses, including TBSV, the extensive replication compartments contain many membranous vesicle-like structures, also called spherules, which are 50–100 nm invaginations with a narrow opening towards the cytosol. Other viruses have membranous, protrusion-type structures with single- or double-membrane structures formed via major membrane rearrangements [1–5]. Regardless of the structure of these replication organelles, it has been proposed that these elaborate membranous structures serve as platforms to assemble VRCs and to concentrate viral and host components for more efficient viral RNA synthesis. In addition, VRCs might also hide the viral RNAs from recognition by the antiviral surveillance system and protect against degradation by cytosolic ribonucleases.

In case of plant and insect viruses, the viral RNA-triggered adaptive innate immune response, called RNAi or RNA silencing response limits viruses to replicate and spread in infected tissues [6–11]. RNAi also contributes to viral RNA recombination and defective viral RNA production [12,13]. The viral double-stranded (ds)RNA replication intermediate or long structured portions of ssRNAs are recognized by the core components of RNAi, which consist of the Dicer-like (DCL) ribonuclease and Argonaute (AGO)-like proteins with RNA slicing activities [7,14–17]. The Dicer-like nucleases process these RNAs into 21–24 nt dsRNAs, called small-interfering siRNAs, via their RNase III activities. Then, the siRNAs are incorporated into the RNA-induced silencing complex (RISC) that contains the AGO proteins. Then, the RISC recognizes the target ssRNAs, followed by slicing and destruction of the target RNA by the RNase H-like activity of AGO [7,8,14]. The core RNAi components and pathways are conserved from some fungi (*Neurospora* and others) and plants to invertebrates and mammals [7,10,18,19].

TBSV is a well-characterized plant (+)RNA virus with one 4.8 kilobase genomic (g)RNA, which codes for only five proteins, two of which are replication proteins, namely p33 and p92^pol^. The p92^pol^ RNA-dependent RNA polymerase (RdRp) is expressed from the gRNA via a translational readthrough mechanism of the p33 stop codon [20–22]. The pre-readthrough protein p33 is an RNA chaperone, and a membrane-associated RNA-binding protein that functions as a master regulator of TBSV replication. Accordingly, p33 plays a role in every step of replication, including in viral RNA recruitment, VRC assembly and RNA synthesis [23–26]. Through its interactions with other p33 and p92 molecules, cellular lipids and 50–100 host proteins, p33 is involved in the formation of the replication compartment [24,27–30]. The tombusvirus VRC contains several host proteins [30–32], including heat shock protein 70 (Hsp70), glyceraldehyde-3-phosphate dehydrogenase (GAPDH), eukaryotic elongation factor 1A (eEF1A), eEF1Bγ, DEAD-box RNA helicases, and the ESCRT (endosomal sorting complexes required for transport) family of host proteins [32–39]. These proteins are required for VRC assembly or affect viral RNA synthesis [27,34,37,40–42]. The TBSV replication process also depends on phospholipids, mainly phosphatidylethanolamine (PE), and sterols, which are actively enriched within the viral replication compartment [43–47].

It is assumed that (+)RNA viruses avoid the powerful RNAi response in plants by forming VRCs and replication compartments hidden away from RNAi and also via expression of suppressors of RNAi, which are part of counter defense strategies against RNAi [9,48,49]. Accordingly, the dsRNA replication intermediate formed during TBSV replication [50], is part of the membrane-bound VRCs. A long-standing unanswered question is that which co-opted host components are involved in VRC assembly that protect (+)RNA viruses during replication from the potent RNAi response. The protection provided by VRCs seems significant, because plant (+)RNA viruses could replicate and accumulate even in the absence of RNAi suppressors [51,52]. Moreover, the Dicer-like enzymes could not completely degrade the viral dsRNA in infected cells, indicating that the viral dsRNA enjoys significant protection during replication.

To answer this fundamental question on the putative role of the co-opted host factors in protecting the viral dsRNA during replication, we took advantage of yeast (*Saccharomyces cerevisiae*), which lacks the RNAi machinery, as a surrogate host for TBSV. Co-expression of a TBSV replicon (rep)RNA with p33 and p92^pol^ replication proteins leads to robust TBSV replication in yeast [53,54], helping the dissection of the roles of subverted host factors in virus replication and virus–host interactions [27,28,55]. We have used the reconstituted RNAi machinery from *S*. *castellii*, which consists of the two-component *DCR1* and *AGO1* genes [56], as a simple, easily tractable system to study the effect of RNAi on TBSV in yeast. Based on this surrogate host system, we show evidence that deletion of a selected group of host factors, namely ESCRT proteins, and alteration of lipid levels, including PE and sterols, greatly affect TBSV accumulation when RNAi activity is induced. Based on our results, we propose that the co-opted host factors are critical for TBSV to assemble membranous VRCs that protect against RNAi activity.

## Results

### Use of the reconstituted RNAi machinery from *Saccharomyces castellii* in *S*. *cerevisiae* as an intracellular probe to measure the accessibility of TBSV RNA during replication

**Fig 1:**
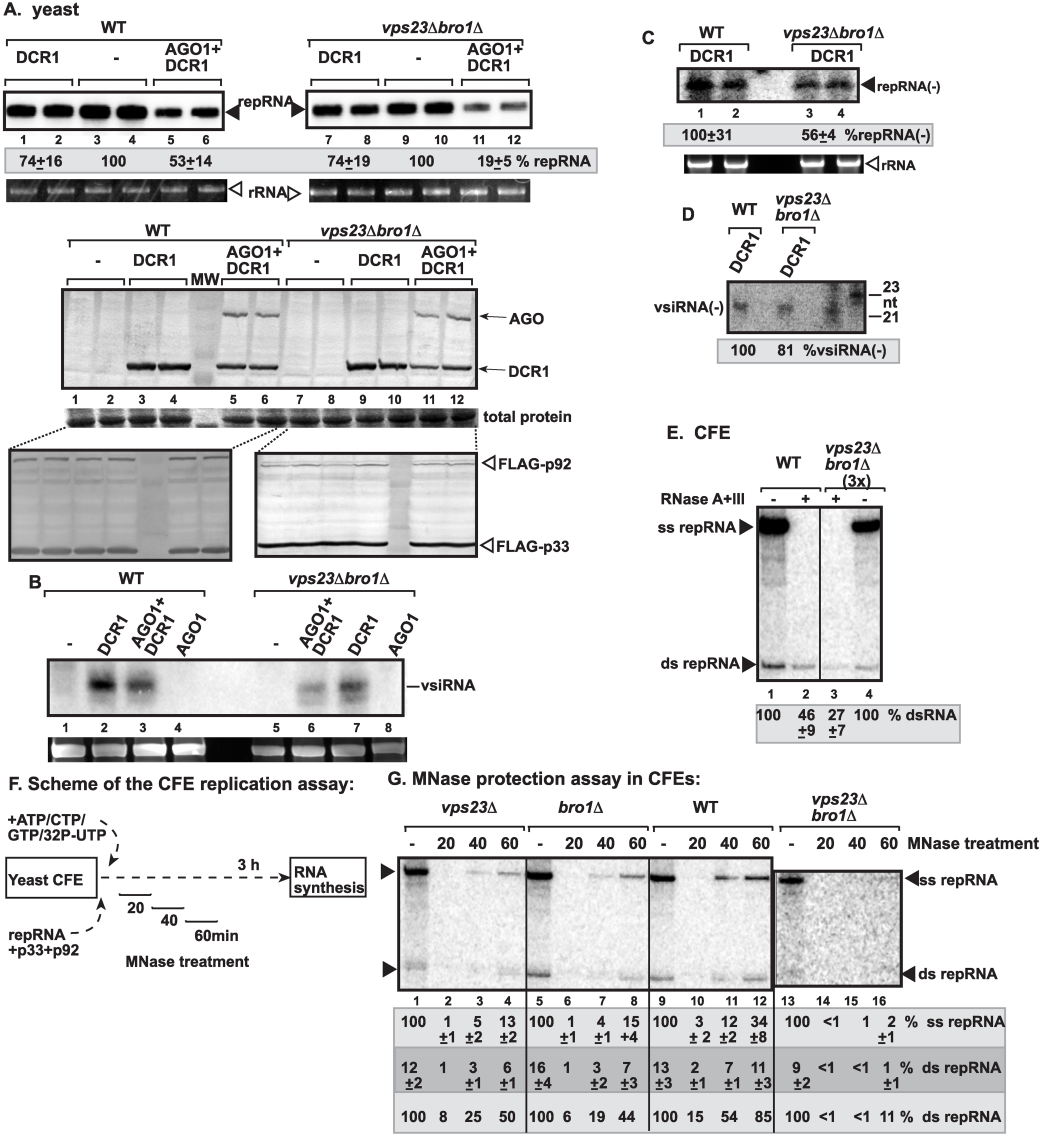
Deletion of ESCRT-I or the ESCRT-accessory Bro1 sensitizes tombusvirus RNA to RNAi-based degradation in yeast. (A) Co-expression of *S*. *castellii* AGO1 and DCR1 in *vps23Δbro1Δ* yeast reduces TBSV repRNA accumulation more than in wt yeast (BY4741). Top panel: Replication of the TBSV repRNA was measured by Northern blotting 24 h after initiation of TBSV replication. The accumulation level of repRNA was normalized based on the ribosomal (r)RNA. Note that the TBSV repRNA, p33 and p92 replication proteins were expressed from plasmids. Each sample is obtained from different yeast colonies. Yeast strain not expressing RNAi components is taken as 100% in each experiment. Average value and standard deviation is calculated from all the biological repeats. Middle and bottom panels: The accumulation levels of His_6_-AGO1 and His_6_-DCR1 and FLAG-p33 and FLAG-p92 replication proteins were tested by Western blotting. Total protein level in different samples is shown on separate SDS-PAGE. Each experiment was repeated twice. Ribosomal RNA is shown as a loading control. (B) Northern blot detection of vsiRNA(+) in *vps23Δbro1Δ* and wt (BY4741) yeast strains replicating TBSV repRNA and expressing AGO1, DCR1 or co-expressing AGO1 and DCR1. ^32^P-labeled TBSV DI-72 (-)RNA was used as a probe. Yeasts used for samples in lanes 1 and 5 lacked the RNAi components. (C) Northern blot detection of repRNA(-) in *vps23Δbro1Δ* and wt (BY4741) yeast strains replicating TBSV repRNA and expressing DCR1. ^32^P-labeled TBSV DI-72 (+)RNA (region III/IV) was used as a probe. (D) Northern blot detection of vsiRNA(-) in *vps23Δbro1Δ* and wt (BY4741) yeast strains replicating TBSV repRNA and expressing DCR1 (as in panel C). ^32^P-labeled RIII/IV of TBSV DI-72 (+)RNA was used as a probe. (E) Non-denaturing PAGE analysis of the ^32^P-labeled TBSV repRNA products obtained in the CFE-based assay. The CFEs were prepared from wt BY4741 or the double deletion *vps23Δbro1Δ* yeast strains. Samples were treated with the ssRNA-specific RNase A and the dsRNA-specific RNase III at the end of the assay to target (+)RNA products and dsRNA replication intermediates. Note that we loaded three times more samples from *vps23Δbro1Δ* CFE to facilitate the visualization of the less abundant dsRNA. (F) Scheme of the CFE-based TBSV replication assay with in vitro reconstituted VRCs. MNase treatment (0.05 U/μl) was performed for 20 min, as shown, followed by inactivation of the MNase with EGTA. Each CFE-based assay lasted for three hours to accomplish maximum level of TBSV repRNA accumulation. (G) Increased sensitivity of viral dsRNA products to nuclease treatment in the *vps23Δbro1Δ* CFE-based TBSV replication assay. Non-denaturing PAGE analysis of the ^32^P-labeled TBSV repRNA products obtained in the CFE-based assay programmed with *in vitro* transcribed TBSV DI-72 (+)repRNA and purified recombinant MBP-p33 and MBP-p92^pol^ replication proteins of TBSV. The CFEs were prepared from BY4741 or the mutant yeast strains. Note that we adjusted the sample loading from CFEs obtained from deletion strains to facilitate the visualization of the less abundant dsRNA. Therefore, the untreated samples are taken as 100% for each CFE-based assays. The dsRNA levels are calculated as % of ssRNA in the untreated samples (in dark gray box), whereas dsRNA levels are calculated as % of dsRNA in the untreated samples (in light gray box, bottom). Each experiment was repeated three times and the data were used to calculate standard deviation.

Many co-opted host components are involved in tombusvirus VRC assembly [27,28,55] and they likely protect TBSV RNA from nucleases and antiviral responses during replication. To test this model, we adapted the simple RNAi machinery from *S*. *castellii* [56] as an intracellular probe to measure if a given co-opted host factor contributes to the protection of the viral RNA. Briefly, constitutive co-expression of *S*. *castellii* DCR1 and AGO1 from *TEF1* promoter led to complete inhibition of TBSV repRNA accumulation in wt yeast (S1A Fig), whereas separate expression of DCR1 and AGO1 did not interfere with TBSV repRNA accumulation, suggesting that co-expression of the two components is required for the RNAi machinery. A more suitable strategy for this research was based on the inducible co-expression of *S*. *castellii* DCR1 and AGO1 from *GAL1* promoter from plasmids, which could be suppressed by the addition of glucose and induced by the addition of galactose to the culture media (Fig 1A, lanes 5–6). In this system, after the induction of the expression of both DCR1 and AGO1, the induced RNAi pathway moderately inhibited viral RNA accumulation in wt yeast (Fig 1A, lanes 1–6), likely due to the protection provided by the membranous VRCs. The accumulation of the expected 23 bp vsiRNA demonstrates the operation of the RNAi machinery in this yeast strains expressing either DCR1 alone or co-expressing DCR1 and AGO1 (S1B and S1C Fig).

### Essential roles of the co-opted Vps23 ESCRT-I and Bro1 accessory factor in protection of the tombusvirus RNA against the RNAi machinery from *Saccharomyces castellii* in *S*. *cerevisiae*

TBSV recruits the cellular ESCRT machinery to deform membranes and build spherules containing VRCs within the replication compartment [57,58]. First, the Vps23p ESCRT-I or Bro1p ESCRT accessory protein are recruited via direct binding to p33 replication proteins, followed by recruitment of the ESCRT-III proteins [38,58,59]. Then, Vps4p AAA ATPase is bound by p33, which likely stabilizes the neck structure of the spherule, to prevent scission and closure of the neck [57].

To test if cellular ESCRT factors required for TBSV-induced spherule formation are important for protecting the viral RNAs from RNAi-based degradation, we launched TBSV replication in *vps23Δbro1Δ* yeast [58]. Since the co-opted ESCRT factors affect the absolute level of TBSV replication in yeast [38,58,59], we calculated the extent of reduction in TBSV RNA protection level in the presence of the RNAi machinery based on the repRNA accumulation in the corresponding yeast strain control, not expressing the RNAi machinery (100%). We found that TBSV repRNA accumulation was inhibited by the RNAi machinery by almost three-times more efficiently in *vps23Δbro1Δ* yeast when compared with wt yeast expressing the full-set of the ESCRT components (Fig 1A, lanes 11–12 versus 5–6). Single expression of DCR1 had only small effect on TBSV RNA accumulation in both yeast strains (Fig 1A). The expressions of both DCR1 and AGO1 were comparable in *vps23Δbro1Δ* and wt yeasts (Fig 1A). Moreover, the expression of the tombusvirus p33 and p92^pol^ replication proteins in *vps23Δbro1Δ* or wt yeasts was not affected by the co-expression of DCR1 and AGO1 (Fig 1A, bottom images), suggesting that enhanced susceptibility of tombusviral RNA in *vps23Δbro1Δ* yeast was likely due to the increased antiviral effect of the reconstituted *S*. *castellii* RNAi machinery on the viral RNAs.

Detection of vsiRNA(+) abundance revealed reduced level in *vps23Δbro1Δ* yeast in comparison with the wt yeast expressing DCR1 (Fig 1B). However, the reduction in vsiRNA(+) abundance is likely due to the reduced target viral RNA level in *vps23Δbro1Δ* yeast, which supports only ~20% TBSV repRNA level in comparison with the wt yeast (lacking the RNAi machinery) [58]. Indeed, comparison of vsiRNA(-) level (generated by DCR1 from dsRNA replication intermediate) revealed that *vps23Δbro1Δ* yeast generated almost as much vsiRNA(-) as the wt yeast did (Fig 1D), whereas the repRNA(-) level (representing the dsRNA replication intermediate of the repRNA) in *vps23Δbro1Δ* yeast was half of the level of repRNA(-) detected in wt yeast (Fig 1C).

To examine if the membranous VRCs in *vps23Δbro1Δ* yeast indeed provide less protection to the viral RNAs, we used cell-free extract (CFE)-based assay in the presence or absence of ribonucleases (the single-stranded ssRNA-specific RNase A and the dsRNA-specific RNase III). The TBSV dsRNA was ~2-fold more sensitive to RNases when CFE was prepared from *vps23Δbro1Δ* yeast in comparison with the CFE from wt yeast (Fig 1E). The TBSV ssRNAs, which are continuously released from VRCs as replication goes on, were fully degraded in both CFE assays.

In a second assay to test the level of protection provided by VRCs, we performed in vitro replicase assembly with purified recombinant viral proteins and (+)repRNA transcripts as schematically shown in Fig 1F, followed by viral RNA replication in the presence of micrococcal nuclease (MNase) to destroy the unprotected viral RNAs. The MNase was added at different time points (as shown) for 20 min and then it was inactivated by EGTA, followed by TBSV repRNA replication on the protected TBSV repRNAs up to 3 hours (Fig 1F). When CFE was prepared from wt yeast, then the VRC partially protected the viral dsRNA [produced by minus-strand synthesis on the (+)RNA template] after 40 min, whereas the protection of viral dsRNA was high after 60 min of incubation (Fig 1G, lanes 11 and 12 versus 9). In contrast, the *in vitro* assembled VRC based on CFE prepared from *vps23Δbro1Δ* yeast did not provide any detectable level of protection after 60 min of incubation. We also tested the VRCs assembled in CFEs prepared from *vps23Δ* or *bro1Δ* yeasts. These MNase protection experiments revealed poor dsRNA protection in CFEs from *vps23Δ* or *bro1Δ* yeasts at both 40 and 60 min time points (Fig 1G). However, the protection of TBSV dsRNA in CFEs from *vps23Δ* or *bro1Δ* yeasts were more significant at the 60 min time point than the lack of dsRNA protection provided in CFE prepared from *vps23Δbro1Δ* yeast (compare lanes 4 and 8 with 16, Fig 1G). These data suggest that due to the partially overlapping roles of Vps23p and Bro1p in supporting the formation of VRCs [58], the CFEs prepared from single deletion yeast strains provided better dsRNA protection *in vitro* than the CFE from double-deletion yeast strain. Altogether, the results from two separate in vitro replication assays with CFEs prepared from *vps23Δbro1Δ* yeast showed that the TBSV dsRNA is not well protected from ribonucleases even after the VRC assembly step, thus indicating that the dsRNA inside the VRCs in the absence of Vps23p and Bro1p is continuously exposed to the RNAi machinery or ribonucleases, likely due to incomplete VRC assembly.

### The critical role of the co-opted ESCRT-III factors in protection of tombusvirus RNA against the RNAi machinery

**Fig 2:**
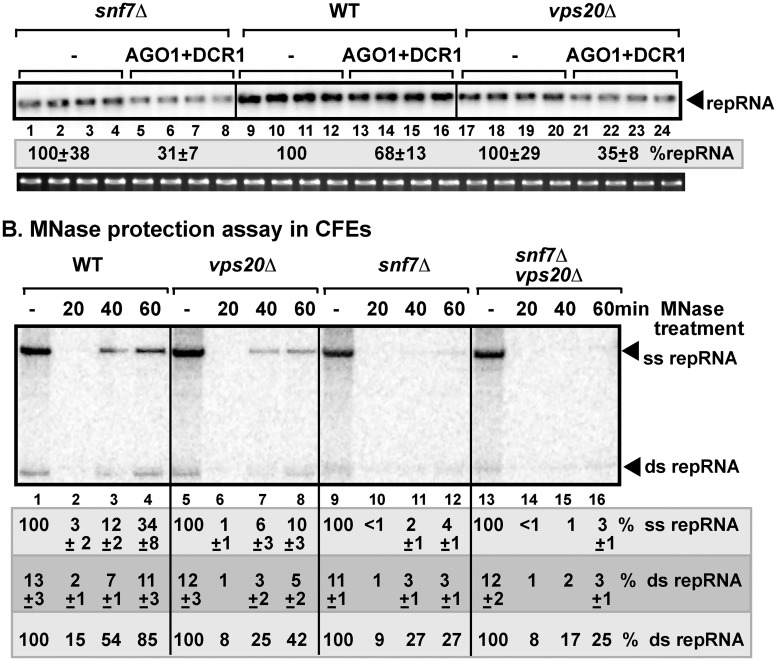
Deletion of ESCRT-III components renders tombusvirus RNA sensitive to co-expression of AGO1 and DCR1 in yeast. (A) Co-expression of *S*. *castellii* AGO1 and DCR1 in *snf7Δ* or *vps20Δ* yeasts reduces TBSV repRNA accumulation more than in wt yeast (BY4741). Top panel: Replication of the TBSV repRNA was measured by Northern blotting 16 h after initiation of TBSV replication. See Fig 1A for further details. (B) Increased sensitivity of viral dsRNA products to nuclease treatment in the *snf7Δ*, *vps20Δ*, or *snf7Δvps20Δ* CFE-based TBSV replication assay. Non-denaturing PAGE analysis of the ^32^P-labeled TBSV repRNA products obtained in the CFE-based assay programmed with *in vitro* transcribed TBSV DI-72 (+)repRNA and purified recombinant MBP-p33 and MBP-p92^pol^ replication proteins of TBSV. See further details in the legends for Fig 1D. Note that the same WT samples are shown here as in Fig 1E, since the same PAGE was used to characterize ESCRT-I and ESCRT-III samples, but they are separately presented for logistic reasons.

Formation of complete vesicle-like structures induced by TBSV in yeast and plants also requires ESCRT-III factors [58]. In the absence of ESCRT-III factors, crescent-like membrane invaginations are formed in yeast replicating TBSV repRNA [58]. To test if Snf7p and Vps20p ESCRT-III factors are important for protecting the viral dsRNA from RNAi-based degradation, we launched TBSV replication either in *snf7Δ* or *vps20Δ* yeasts. We found that TBSV repRNA accumulation was reduced by ~2-fold more in both *snf7Δ* and *vps20Δ* yeasts when AGO1 and DCR1 were co-expressed in comparison with the wt yeast (Fig 2A). Similarly, the dsRNA replication intermediate was protected from MNase treatment by 2-to-4-fold less effectively by the *in vitro* assembled TBSV replicases prepared with CFEs from *snf7Δ* and *vps20Δ* yeasts than by the CFE from WT yeast (Fig 2B). The CFE-based replication assay using the double-deletion strain (*snf7Δvps20Δ* yeast) revealed that the level of dsRNA protection was comparable to that provided by the CFE from single deletion strain (*snf7Δ* yeast) against MNase treatment (Fig 2B), suggesting that the ESCRT-III factors do not have complementary roles in protecting the viral RNA from RNAi activities. Altogether, the results from the in vitro replication assay with CFE prepared from single deletion or *snf7Δvps20Δ* yeasts indicated that the dsRNA inside the VRCs in the absence of Snf7p or Vps20p ESCRT-III factors is continuously exposed to the RNAi machinery or ribonucleases, likely due to incomplete VRC assembly.

### Co-opted sterols and oxysterol-binding proteins play a role in protection of tombusvirus RNA against the RNAi machinery

**Fig 3:**
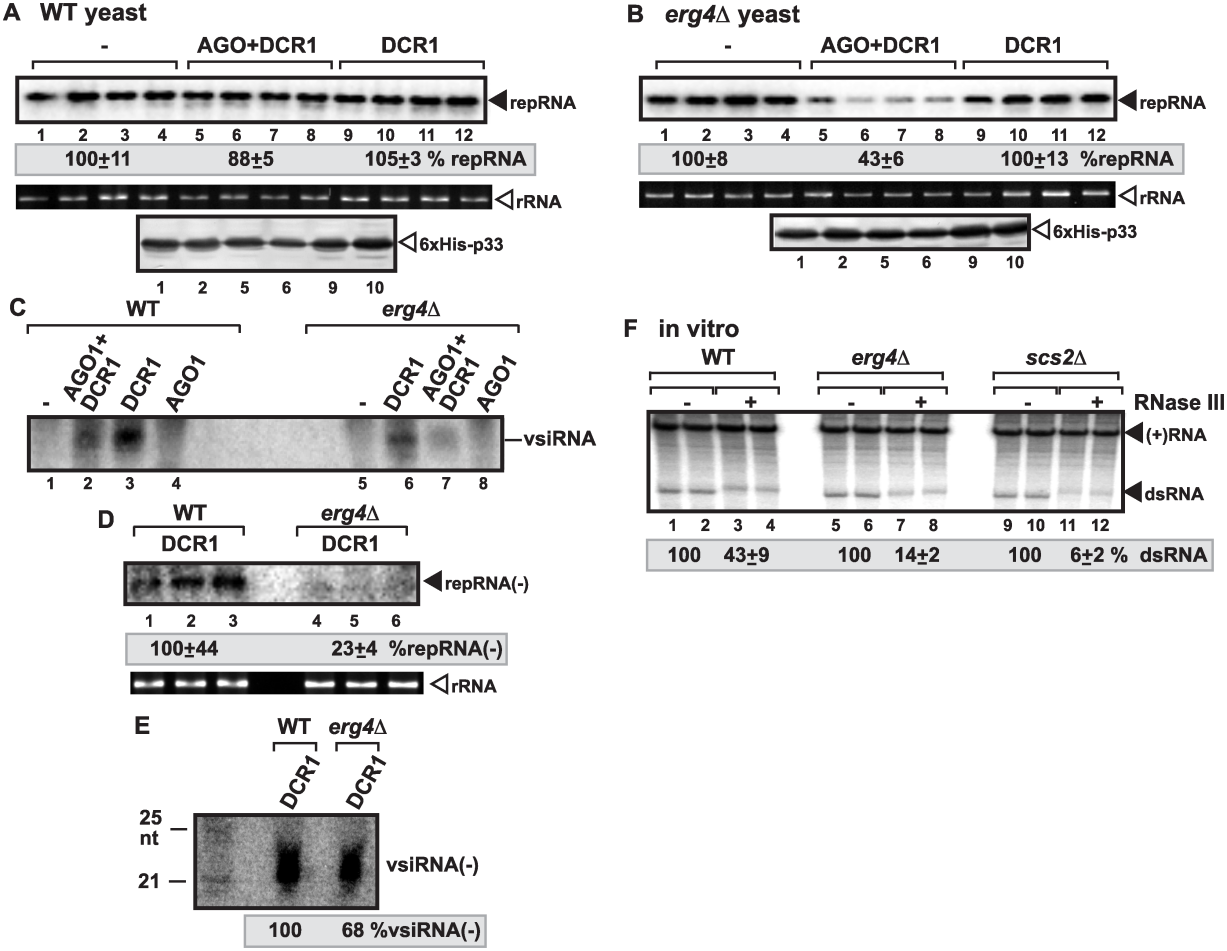
Reduced ergosterol biosynthesis in yeast increases the sensitivity of replicating tombusvirus RNA to the reconstituted RNAi. (A-B) Induction of RNAi in *erg4Δ* yeast inhibits TBSV repRNA accumulation. Top panels: Replication of the TBSV repRNA was measured by Northern blotting 24 h after initiation of TBSV replication in *erg4Δ* and wt BY4741 yeasts. See Fig 1A for further details. (C) Northern blot detection of vsiRNA(+) in *erg4Δ* and wt (BY4741) yeast strains replicating TBSV repRNA and expressing AGO1, DCR1 or co-expressing AGO1 and DCR1. ^32^P-labeled TBSV DI-72 (-)RNA was used as a probe. Yeasts used for samples in lanes 1 and 5 lacked the RNAi components. (D) Northern blot detection of repRNA(-) in *erg4Δ* and wt (BY4741) yeast strains replicating TBSV repRNA and expressing DCR1. ^32^P-labeled region III/IV of TBSV DI-72 (+)RNA was used as a probe. (E) Northern blot detection of vsiRNA(-) in *erg4Δ* and wt (BY4741) yeast strains replicating TBSV repRNA and expressing DCR1 (as in panel D). ^32^P-labeled RIII/IV of TBSV DI-72 (+)RNA was used as a probe. (F) Non-denaturing PAGE analysis of the ^32^P-labeled TBSV repRNA products obtained in the CFE-based assay. The CFEs were prepared from wt BY4741, *erg4Δ*, or *scs2Δ* yeast strains, which co-expressed p33 and p92 replication proteins, and programmed with DI-72 (+)repRNA transcripts. Samples were treated with the dsRNA-specific RNase III during the entire assay to target accessible dsRNA replication intermediates.

Formation of tombusvirus VRCs is greatly affected by sterols *in vitro*, in yeast and plants [45,47]. The co-opted sterols, which are enriched within the replication compartment [45], likely enhance the stability of vesicle-like structures and facilitate tighter packing of phospholipids in the membranes used by TBSV for VRC assembly. These features of sterols might also contribute to the protection of viral dsRNA replication intermediate provided by the membranous VRCs against cellular nucleases. This theory was first tested using a yeast strain deficient in ergosterol (yeast version of cholesterol) biosynthesis due to deletion of C-24 sterol reductase (*erg4Δ* yeast) [47,60]. Induction of the RNAi machinery in *erg4Δ* yeast inhibited TBSV repRNA accumulation by ~2-fold more efficiently than in wt yeast (Fig 3A and 3B).

Detection of vsiRNA(+) abundance revealed reduced level in *erg4Δ* yeast in comparison with the wt yeast expressing DCR1 (Fig 3C). However, the reduction in vsiRNA(+) abundance is likely due to the reduced target viral RNA level in *erg4Δ* yeast, which supports only ~20% TBSV repRNA level in comparison with the wt yeast (lacking the RNAi machinery) [60]. Therefore, we also measured vsiRNA(-) level (generated by DCR1 from dsRNA replication intermediate), which showed only slight reduction in *erg4Δ* yeast in comparison with vsiRNA(-) level in wt yeast (Fig 3E). However, the repRNA(-) level (representing the dsRNA replication intermediate of the repRNA) in *erg4Δ* yeast was only ~20% of the level of repRNA(-) detected in wt yeast (Fig 3D), which is comparable to the reduction in repRNA(+) level in *erg4Δ* versus wt yeasts [60]. Thus, the vsiRNA(-) was generated from 5-fold less dsRNA templates by DCR1 in *erg4Δ* yeast than in wt yeast, suggesting that DCR1 could produce vsiRNA(-) in ~3-fold higher ratio in *erg4Δ* yeast than in wt yeast.

Moreover, the CFE-based TBSV replication assay showed poor protection of TBSV dsRNA against the dsRNA-specific RNase III within VRCs assembled in *erg4Δ* yeast in comparison with wt yeast (Fig 3F). This suggests that the dsRNA within the VRCs formed in sterol-depleted cells is continuously exposed to RNAi/ribonucleases.

**Fig 4:**
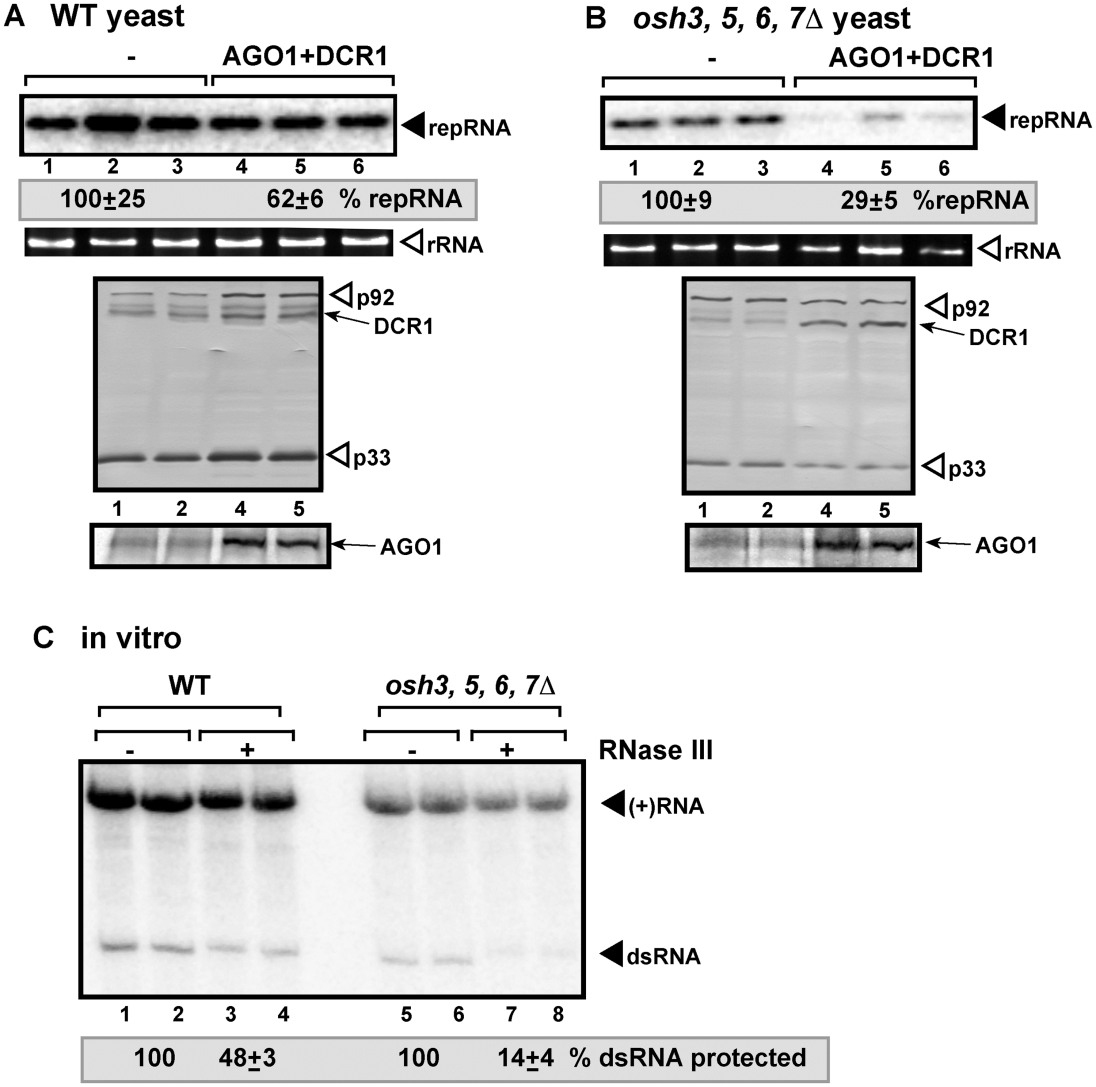
Deletion of ORP genes renders tombusvirus RNA sensitive to the reconstituted RNAi in yeast. (A-B) Induction of RNAi in *osh3*,*5*,*6*,*7Δ* yeast inhibits TBSV repRNA accumulation. Top panels: Replication of the TBSV repRNA was measured by Northern blotting 16 h after initiation of TBSV replication in wt SEY6210 or *osh3*,*5*,*6*,*7Δ* yeast strains. See Fig 1A for further details. (C) Non-denaturing PAGE analysis of the ^32^P-labeled TBSV repRNA products obtained in the CFE-based assay. The CFEs were prepared from wt SEY6210 or *osh3*,*5*,*6*,*7Δ* yeast strains, which co-expressed p33 and p92 replication proteins, and programmed with DI-72 (+)repRNA transcripts. Samples were treated with the dsRNA-specific RNase III during the entire assay to target accessible dsRNA replication intermediates.

To further confirm the critical role of the co-opted sterols in the formation of more ribonuclease resistant VRCs, we also studied the effect of co-opted cellular proteins involved in membrane-contact site (MCS) formation [45]. The tombusvirus-induced MCSs are formed between the ER and peroxisome membranes, when the two organellar membranes are juxtaposed, with the help of co-opted ER-resident VAP proteins (Scs2 in yeast, see below) and the oxysterol-binding proteins (OSBP-related protein or ORP). The tombusvirus-induced MCSs help the enrichment of sterols within the replication compartment. First, we targeted four members of the oxysterol-binding proteins, namely Osh3, 5, 6 and 7, which are recruited by TBSV via interaction with p33 replication proteins to the site of replication. These ORP/Osh proteins are involved in the formation of MCSs and enrichment of sterols within the replication compartment [45]. Induction of AGO1 and DCR1 in *osh3*,*5*,*6*,*7Δ* yeast inhibited TBSV repRNA accumulation by ~2-fold more efficiently than in wt yeast (Fig 4A and 4B). In addition, the CFE-based *in vitro* replication assay revealed that the VRCs assembled in *osh3*,*5*,*6*,*7Δ* yeast could not protect efficiently the TBSV dsRNA intermediate against RNase III (Fig 4C). Second, we tested the ER-resident Scs2p VAP, which is also recruited by TBSV via interaction with p33 replication proteins to help the formation of MCSs and enrichment of sterols within the replication compartment [45,61]. Induction of AGO1 and DCR1 in *scs2Δ* yeast led to ~3-fold decreased TBSV repRNA accumulation than in the absence of RNAi, while the RNAi was less efficient in the parental yeast strain carrying wt copy of *SCS2* (S2 Fig). The VRCs assembled in *scs2Δ* yeast provided negligible level of protection against RNase III in the in vitro replication assay (Fig 3F and S2C Fig). Overall, reduction of sterol biosynthesis (in *erg4Δ* yeast) or inhibition of virus-induced MCS formation (in *osh3*,*5*,*6*,*7Δ* or *scs2Δ* yeasts) that hinders the local sterol enrichment at replication sites greatly inhibited the assembly of RNase-resistant VRCs in yeast or *in vitro*. Thus, co-opted sterols play important roles in formation of RNAi-resistant replication compartment.

### Phosphatidylethanolamine level affects the formation of RNAi-resistant replication compartment

Tombusvirus replication greatly depends on phospholipid levels, especially on phosphatidylethanolamine (PE), which is highly enriched within the replication compartment [43]. PE is required for TBSV replication in an artificial vesicle- (liposome)-based *in vitro* assay [43] and it enhances the activation of the p92 RdRp in vitro [62]. Moreover, the PE level is increased during TBSV replication in yeast and plant cells and high PE level in yeast via modulation of phospholipid biogenesis genes also leads to enhanced TBSV replication [43,44].

**Fig 5:**
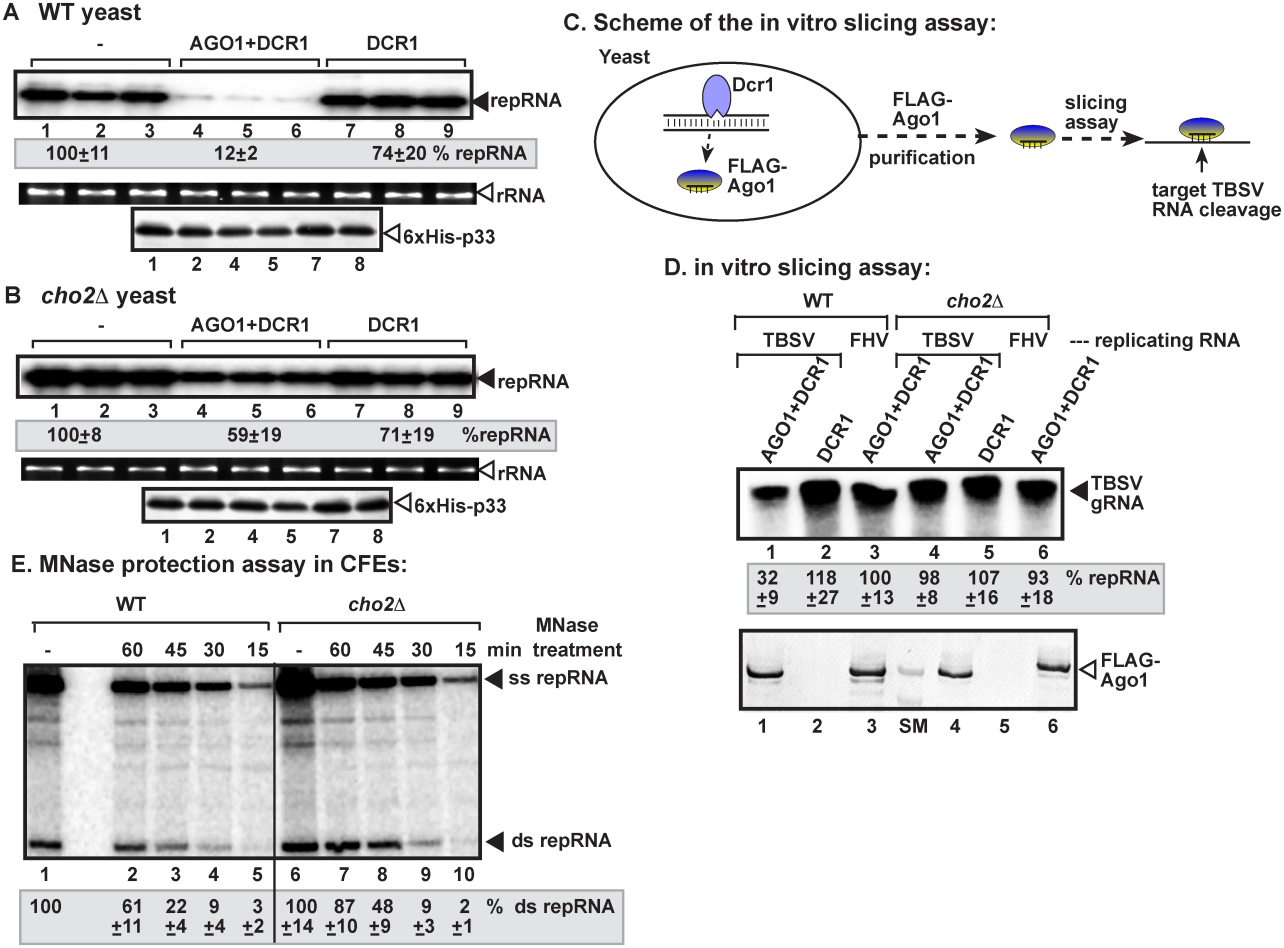
Elevated PE level in yeast decreases the sensitivity of replicating tombusvirus RNA to the reconstituted RNAi. (A-B) Induction of RNAi in *cho2Δ* yeast inhibits TBSV repRNA accumulation less effectively than in wt yeast. Top panels: Replication of the TBSV repRNA was measured by Northern blotting 16 h after initiation of TBSV replication in wt BY4741 or *cho2Δ* yeast strains. Note that AGO1 and DCR1 were continuously expressed during the entire yeast culturing process to enhance RNAi activities in both strains. See Fig 1A for further details. (C) A scheme of the in vitro slicing assay. FLAG-tagged AGO1 was affinity-purified from yeasts replicating either TBSV repRNA or the unrelated FHV RNA1 and co-expressing FLAG-AGO1 and DCR1. The purified FLAG-AGO1 was then used to cleave the target ^32^P-labeled TBSV genomic RNA. We could not detect the in vitro slicing products on the gels likely due to the multiple cleavages and at different positions of the target RNAs. (D) In vitro slicing activity of AGO1 purified from *cho2Δ* yeast is reduced in comparison with that of AGO1 from wt yeast. Top image: Denaturing PAGE analysis of the products from the in vitro slicing assay. Preparations obtained from yeasts expressing DCR1 only were used as a negative control. Bottom panel: Immuno-blot detection of affinity-purified FLAG-AGO1 with anti-FLAG antibody in samples used in the in vitro slicing assay above. (E) Decreased sensitivity of viral dsRNA products to MNase treatment (0.05 U/μl) in the *cho2Δ* CFE-based TBSV replication assay. Non-denaturing PAGE analysis of the ^32^P-labeled TBSV repRNA products obtained in the CFE-based assay programmed with *in vitro* transcribed TBSV DI-72 (+)repRNA and purified recombinant MBP-p33 and MBP-p92^pol^ replication proteins of TBSV. The CFEs were prepared from wt BY4741 or *cho2Δ* yeast strains. MNase treatment was performed for 15 min, as shown, followed by inactivation of the MNase with EGTA. Each CFE-based assay lasted for three hours to accomplish maximum level of TBSV repRNA accumulation. See further details in the legends for Fig 1D and 1E. Each experiment was repeated.

To test the role of PE-rich membranes in the protection of the viral dsRNA from RNAi, we induced the RNAi machinery in *cho2Δ* yeast, which is partially defective in converting PE to PC, thus leading to elevated PE level and enhanced TBSV replication [43]. Since the VRCs formed in wt yeast could provide good level of protection against RNAi under our standard conditions (Figs 1–4), we applied constitutive expression of the RNAi machinery (AGO1 and DCR1) that could enhance the effectiveness of RNAi in these experiments. This is achieved by culturing yeast in media supplemented with galactose all the time (resulting in constitutive expression of both DCR1 and AGO1). Under these conditions, the VRCs assembled in wt yeast provided less protection (Fig 5A, lanes 4–6). On the contrary, the level of TBSV dsRNA protection was ~5-fold higher in *cho2Δ* yeast expressing AGO1 and DCR1 (Fig 5B, lanes 4–6), suggesting that increased PE levels provide more protective subcellular environment for VRC assembly.

To further test if the viral RNA is less accessible in *cho2Δ* yeast to the RNAi machinery than in wt yeast, we utilized an in vitro slicing assay based on purification of FLAG-tagged AGO1 from *cho2Δ* and wt yeasts replicating TBSV repRNA and co-expressing DCR1 and FLAG-AGO1. In these yeasts, AGO1 is expected to be loaded with vsiRNA, which then could activate AGO1 endonuclease activity specifically against TBSV RNA target in vitro (Fig 5C). By using comparable amounts of purified FLAG-AGO1 preloaded with vsiRNA, we tested the slicing activity on ^32^P-labeled TBSV RNA in vitro. The purified FLAG-AGO1 from wt yeast replicating the TBSV repRNA showed slicing activity against ^32^P-labeled TBSV gRNA (Fig 5D, lane 1), whereas the purified FLAG-AGO1 from wt yeast replicating the unrelated Flock House virus RNA1 did not have a slicing activity against ^32^P-labeled TBSV gRNA (Fig 5D, lane 3), thus confirming the presence of bona fide RNAi machinery in wt yeast co-expressing AGO1 and DCR1. On the contrary, the purified FLAG-AGO1 from *cho2Δ* yeast replicating TBSV repRNA showed negligible slicing activity against ^32^P-labeled TBSV gRNA (Fig 5D, lane 4), suggesting that either DCR1 or AGO1 had a limited access to the TBSV replication compartment.

To define how increased PE level provides better protection against ribonucleases, we performed CFE-based replication/protection assay using purified recombinant p33/p92 for *de novo* assembly of VRCs in CFEs prepared from *cho2Δ* versus wt yeasts (Fig 5E). This assay can test the speed of VRC assembly in vitro, based on measurement of the level of dsRNA protection provided by VRCs against MNase, which was added at various time points (Fig 5E). When CFE was prepared from wt yeast, then the in vitro assembled VRCs partially protected the viral dsRNA after 45 min, and ~60% after 60 min of incubation (Fig 5E, lanes 2 and 3 versus 1). On the other hand, CFE prepared from *cho2Δ* yeast showed some dsRNA protection as early as 30 min and high level of protection by 45 min and complete protection by 60 min of incubation prior to MNase treatment (Fig 5E, lanes 7–9 versus 6). Thus, we suggest that accelerated VRC assembly due to high PE level in *cho2Δ* yeast might decrease the time available for ribonucleases to associate with VRCs during their assembly process.

### Level of phospholipid synthesis affects the formation of RNAi-resistant replication compartment

**Fig 6:**
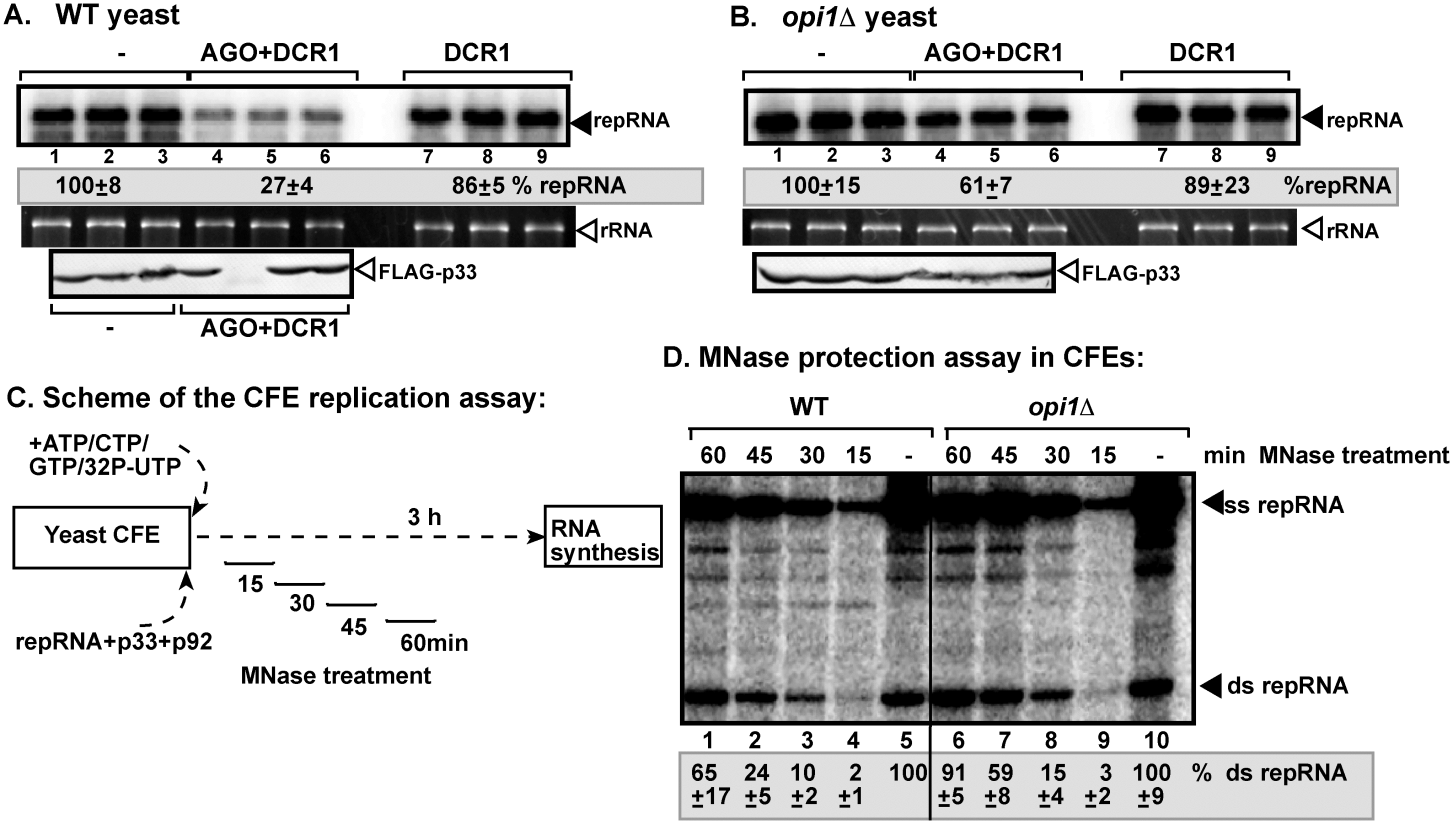
Elevated phospholipid level in yeast decreases the sensitivity of replicating tombusvirus RNA to the reconstituted RNAi. (A-B) Induction of RNAi in *opi1Δ* yeast inhibits TBSV repRNA accumulation less effectively than in wt yeast. Top panels: Replication of the TBSV repRNA was measured by Northern blotting 24 h after initiation of TBSV replication in wt BY4741 or *opi1Δ* yeast strains. Note that AGO1 and DCR1 were continuously expressed during the entire yeast culturing process to enhance RNAi activities in both strains. See Fig 1A for further details. (C) Scheme of the CFE-based TBSV replication assay with in vitro reconstituted VRCs. MNase treatment (0.05 U/μl) was performed for 15 min, as shown, followed by inactivation of the MNase with EGTA. Each CFE-based assay lasted for three hours to accomplish maximum level of TBSV repRNA accumulation. (D) Decreased sensitivity of viral dsRNA products to MNase treatment (0.05 U/μl) in the *opi1Δ* CFE-based TBSV replication assay. Non-denaturing PAGE analysis of the ^32^P-labeled TBSV repRNA products obtained in the CFE-based assay programmed with *in vitro* transcribed TBSV DI-72 (+)repRNA and purified recombinant MBP-p33 and MBP-p92^pol^ replication proteins of TBSV. The CFEs were prepared from BY4741 or *opi1Δ* yeast strains. Each experiment was repeated.

The overall phospholipid content in yeast could be increased by deleting *OPI1*, which is a repressor of expression of many phospholipid synthesis genes [63]. The higher level of phospholipids favors TBSV repRNA accumulation by providing easy access to membranes for VRC assembly [46,64]. TBSV repRNA accumulation reached ~2-fold higher level in *opi1Δ* yeast than in WT yeast expressing AGO1 and DCR1 constitutively (Fig 6B, lanes 4–6), suggesting that increased phospholipid levels facilitate VRC assembly in a more protective subcellular environment.

The CFE prepared from *opi1Δ* yeast supported VRC assembly that provided high level of dsRNA protection by 45 min and complete protection by 60 min of incubation prior to MNase treatment (Fig 6C and 6D, lanes 6–7 versus 10). Whereas, CFE prepared from wt yeast supported the in vitro VRC assembly at a slower pace as the viral dsRNA was only partially protected after 45 min, and at a ~60% level after 60 min of incubation (Fig 6D, lanes 1 and 2 versus 5). These data suggest that the VRC assembly was enhanced due to increased level of phospholipids in *opi1Δ* yeast, possibly leading to reduced time available for ribonucleases to associate with VRCs during their assembly process.

On the contrary, decreasing cellular phospholipid levels via deletion of *INO2* transcription factor required for expression of many phospholipid synthesis genes [63], resulted in ~3-fold more reduction in repRNA level when AGO1 and DCR1 were expressed in *ino2Δ* yeast in comparison with wt yeast (supplement S3 Fig). In addition, the CFE preparation obtained from *ino2Δ* yeast provided poor dsRNA protection in vitro against RNase III (S3 Fig, panel C, lanes 7–8 versus 5–6). Thus, these data indicate that inhibition of phospholipid synthesis likely reduces the formation of RNAi-resistant replication compartment.

### Forcing TBSV replication to utilize ER membranes leads to the formation of RNAi-resistant replication compartment

**Fig 7:**
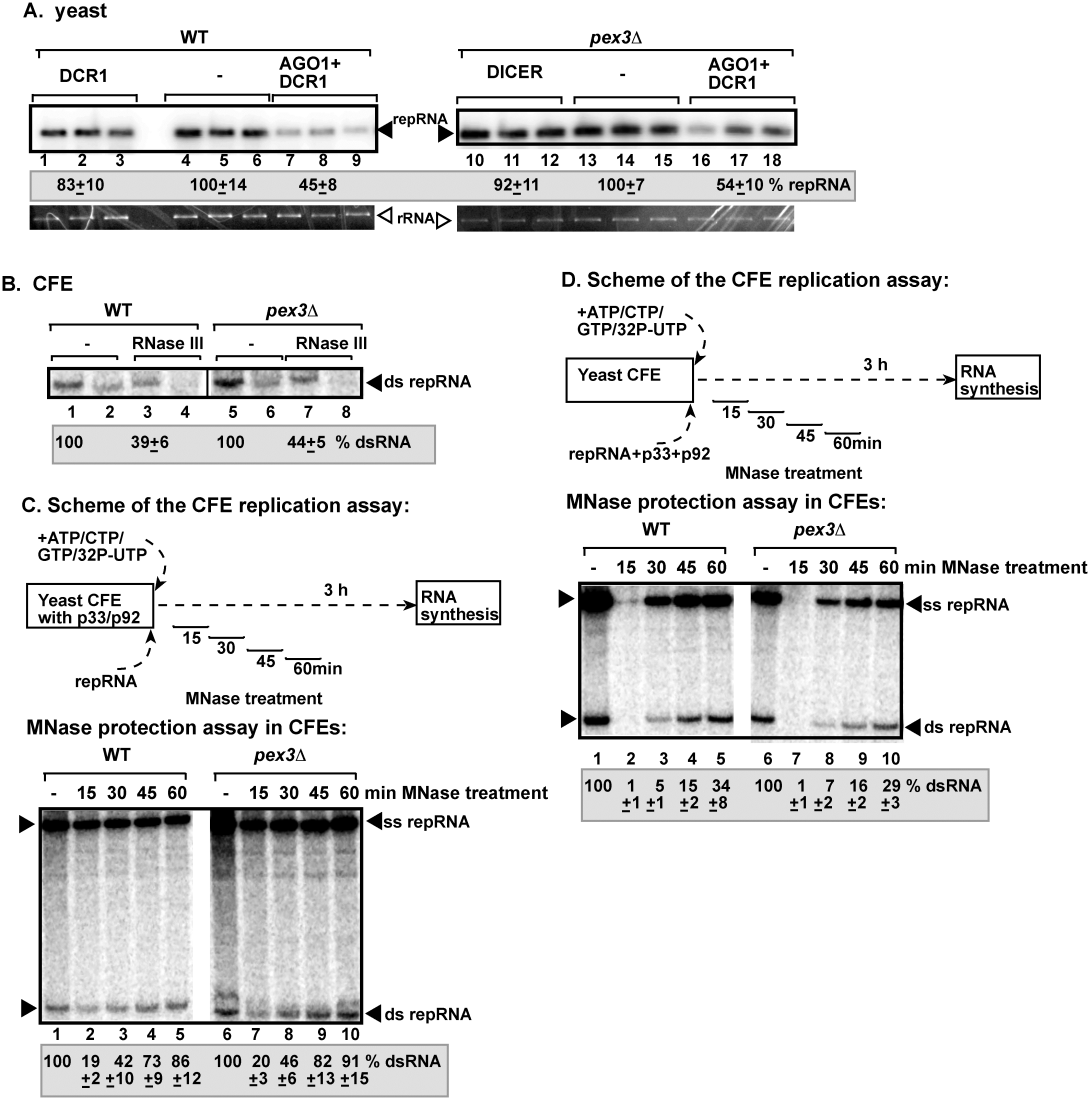
TBSV generates RNAi-insensitive compartments as efficiently in the ER as in the peroxisomes. (A) Deletion of *PEX3* gene results the switch of TBSV replication to the ER, yet the VRCs assembled on the ER membrane show comparable protection against RNAi as those assembled on the peroxisomal membranes. Replication of the TBSV repRNA was measured by Northern blotting 16 h after initiation of TBSV replication in wt BY4741 or *pex3Δ* yeasts. See Fig 1A for further details. (B) Non-denaturing PAGE analysis of the ^32^P-labeled TBSV repRNA products obtained in the CFE-based assay. The CFEs were prepared from wt BY4741 or *pex3Δ* yeast strains, which co-expressed p33 and p92 replication proteins, and programmed with DI-72 (+)repRNA transcripts. Samples were treated with the dsRNA-specific RNase III during the entire assay to target accessible dsRNA replication intermediates. Samples 2, 4, 6 and 8 were heat treated to denature dsRNA products, whereas samples 1, 3, 5 and 7 were not heat-treated. (C) Comparable sensitivity of the viral dsRNA products to MNase treatment in the *pex3Δ* CFE-based to wt CFE-based TBSV replication assay. Top: Scheme of the CFE-based TBSV replication assay. MNase treatment (0.1 U/μl) was performed for 15 min, as shown, followed by inactivation of the MNase with EGTA. Each CFE-based assay lasted for three hours to accomplish maximum level of TBSV repRNA accumulation. Bottom: Non-denaturing PAGE analysis of the ^32^P-labeled TBSV repRNA products obtained in the CFE-based assay. Each experiment was repeated. (D) Top: Scheme of the CFE-based TBSV replication assay with in vitro reconstituted VRCs. The CFEs were prepared from wt BY4741 or *pex3Δ* yeast strains and programmed with *in vitro* transcribed TBSV DI-72 (+)repRNA and purified recombinant MBP-p33 and MBP-p92^pol^ replication proteins of TBSV. Bottom: Non-denaturing PAGE analysis of the ^32^P-labeled TBSV repRNA products obtained in the CFE-based assay. See further details in panel C. Each experiment was repeated.

One of the remarkable features of TBSV replication is that it can switch to the ER membranes when the peroxisomes are absent in yeast due to deletion of either *PEX3* or *PEX19* peroxisome membrane biogenesis genes [65]. To test if the VRCs formed by usurping ER membranes are RNAi insensitive, we co-expressed AGO1 and DCR1 in *pex3Δ* and wt yeasts, respectively, replicating TBSV repRNA. The accumulation of repRNA was comparable in these yeasts (Fig 7A). In addition, the CFE preparation obtained from *pex3Δ* yeast provided comparable level of dsRNA protection against RNase III to the CFE prepared from wt yeast (Fig 7B, lanes 3 versus 7).

In the second assay to test the level of dsRNA protection provided by VRCs formed using the ER membranes, we performed in vitro replication using CFEs prepared from wt and *pex3Δ* yeast with pre-expressed viral proteins (Fig 7C). These CFEs were programmed with (+)repRNA transcripts, followed by viral RNA replication in the presence of MNase to destroy the unprotected viral RNAs. The MNase was added at different time points (as shown) for 15 min and then the MNase was inactivated by EGTA, followed by TBSV repRNA replication based on the protected TBSV repRNAs up to 3 hours (Fig 7C). The level of protection of the viral dsRNA provided by the VRCs was comparable after 60 min of incubation in wt and *pex3Δ* CFEs (Fig 7C, lanes 5 versus 10). Also, the kinetics of VRC assembly, based on the level of protection of the viral dsRNA provided by the VRCs, was comparable at four different time points in wt and *pex3Δ* CFEs (Fig 7C).

To further confirm these findings, we performed a third assay, in which the CFEs prepared from wt and *pex3Δ* yeasts (lacking viral components) were programmed with purified recombinant p33 and p92 replication proteins and (+)repRNA transcripts as depicted in Fig 7D. The level of protection of the viral dsRNA provided by the VRCs against MNase treatment was comparable in all four time points tested in wt and *pex3Δ* CFEs (Fig 7D). These results indicate that the subverted ER membranes in *pex3Δ* could provide as good protection for TBSV dsRNA against RNAi or ribonucleases as the peroxisomal membranes in wt yeast.

## Discussion

In this paper, we have used a reconstituted antiviral defense pathway in a model host system to further our understanding of virus-host interactions in general, and specifically the role of the membranous VRC in protection of the viral dsRNA replication intermediates against ribonucleases. This is based on the RNAi machinery of *S*. *castellii*, which only consists of the two-component *DCR1* and *AGO1* genes [56]. This simple RNAi machinery is known to be effective against the yeast L-A dsRNA virus and its satellite RNA in yeast [66], and TBSV (this work). Similar to the RNAi machinery of higher eukaryotes [9], the reconstituted RNAi machinery of *S*. *castellii* also requires both DCR1 and AGO1 proteins to be effective against TBSV. DCR1 produces 23 bp vsiRNA [66], as we also observed in case of TBSV. We found that this reconstituted antiviral defense pathway in surrogate host yeast is useful as an intracellular probe to further our understanding of virus-host interactions and the role of co-opted host factors in formation of membrane-bound viral replicase complexes in protection of the viral RNA against ribonucleases.

We have obtained evidence that a group of pro-viral cellular factors involved in VRC formation is essential for the generation of the protective membranous subcellular environment for TBSV in cells. Previous works with TBSV have shown that tombusviruses co-opt several host factors to build replication compartment required for replication. The replication compartment consists of mostly peroxisomal membranes and includes many virus-induced spherules, which harbor the VRCs [67]. Spherule formation requires the viral replication proteins, the viral (+)RNA and co-opted ESCRT proteins [57,58] that are involved in bending the boundary membranes of peroxisomes or ER membranes towards the organellar lumen [67]. While spherule formation is inhibited when Vps23 ESCRT-I component is missing in yeast, incomplete spherule-like structures with wide openings are formed when ESCRT-III or Vps4 AAA ATPase are deleted in yeast [57,58].

**Fig 8:**
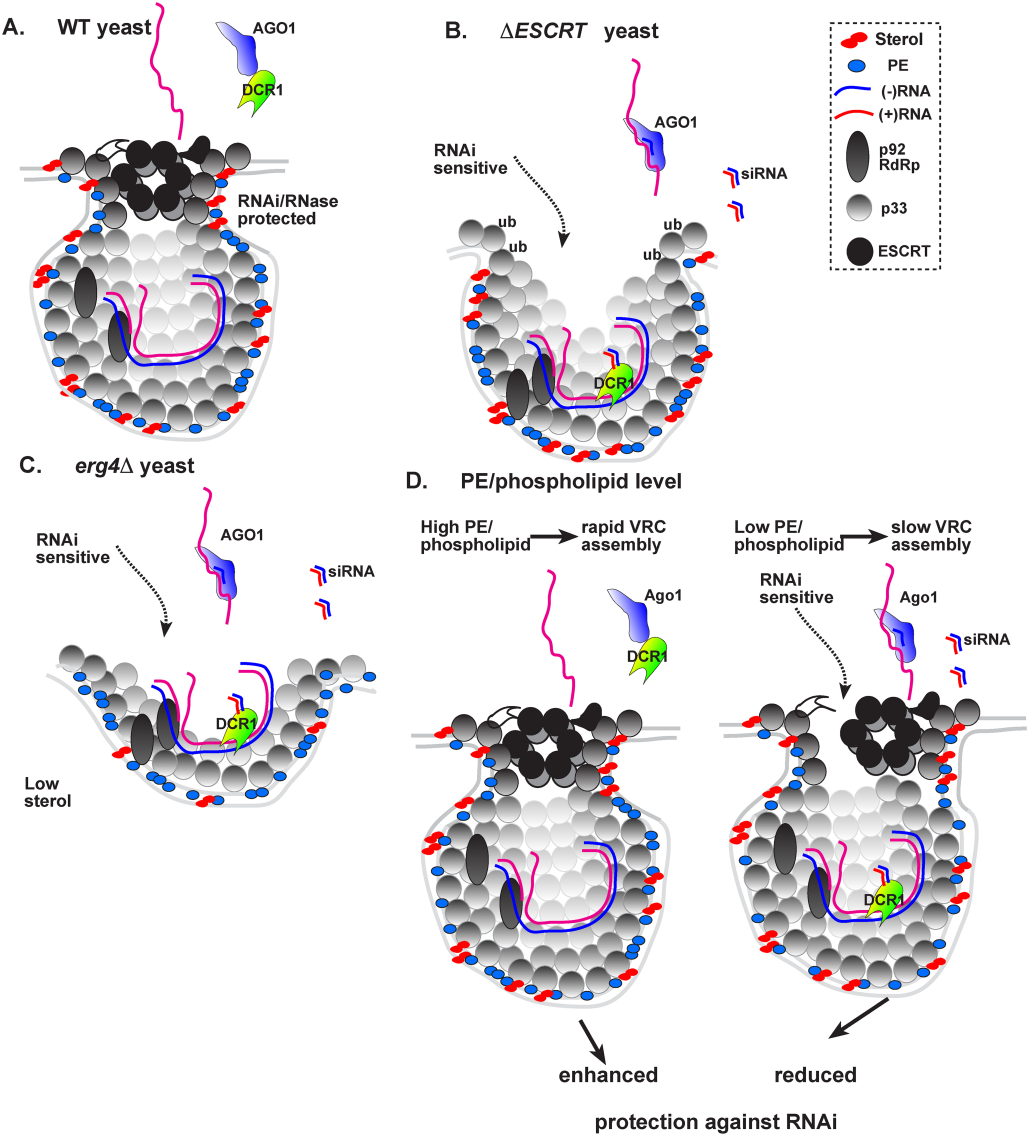
A model on the role of co-opted ESCRT factors and lipids in formation of RNAi-insensitive VRCs during TBSV replication in yeast. (A) In wt yeast, the formation of tombusvirus-induced vesicle-like spherules (membrane invaginations) in the peroxisomal membranes is facilitated by the recruitment of the ESCRT proteins and enrichment of sterols and PE. The model predicts that the membranous VRC protects the viral dsRNA replication intermediate from DCR1, resulting in lack of siRNA generation and the increased stability of both newly-made TBSV (+)RNA and dsRNA replication intermediate, as shown. Note that ESCRT factors have been placed putatively in the VRCs. (B) In the absence of ESCRT-I or III proteins, tombusvirus replication proteins could only form membrane invaginations with large openings that make the viral dsRNA replication intermediate in VRCs exposed to DCR1, resulting in siRNA generation and the decreased stability of both newly-made TBSV (+)RNA and dsRNA replication intermediate. (C) In the absence of TBSV-induced sterol (shown here for ergosterol) enrichment within the replication compartment, the VRCs formed are less stable and the viral dsRNA replication intermediates in VRCs are exposed to DCR1, resulting in siRNA generation and the decreased stability of both newly-made TBSV (+)RNAs and dsRNA replication intermediate. (D) PE and phospholipid levels affect the speed of RNAi-insensitive VRC assembly. Left panel: when the VRC assembly is rapid due to high PE or phospholipid level in yeast, then components of RNAi machinery or ribonucleases have greatly reduced chance to enter the VRCs prior to the completion of VRC assembly. Right panel: when the VRC assembly is slow due to a limiting host factor (such as PE and phospholipid accessibility), then components of RNAi machinery (as indicated by the presence of DCR1 inside the spherule in the right panel) or ribonucleases might be able to enter the VRCs prior to the completion of VRC assembly. This deficiency in excluding ribonucleases from VRCs during slow VRC assembly process then leads to lower level of viral RNA accumulation.

One major proposed function of spherule formation is the protection of the viral dsRNA replication intermediate formed during TBSV replication (Fig 8A) [50]. How much extent is the viral dsRNA, which is present within the membrane-bound VRC, accessible to DCR1 and the RNAi machinery? We found that in comparison with wt yeast expressing the full set of ESCRT factors, deletions of Vps23 ESCRT-I and Bro1 accessory ESCRT protein, which play partial overlapping roles in TBSV replication [58], have rendered the viral dsRNA replication intermediate highly sensitive to the RNAi machinery in yeast and to nucleases *in vitro*. Similarly, deletions of *SNF7* or *VPS20* ESCRT-III factors made TBSV replication more sensitive to AGO1 and DCR1 expression in yeast. Moreover, the lack of Snf7p or Vps20p in combination with Snf7p in CFEs used for assembly of the TBSV replicase *in vitro* resulted in destruction of dsRNA replication intermediate by MNase at the 60 min time point when the CFE from wt yeast has provided good protection for dsRNA (Fig 2). Altogether, these results unambiguously demonstrate that co-opted protein factors, namely the ESCRT factors, are exploited by tombusviruses not only for pro-viral functions to facilitate replication [38,57,58,68], but the ESCRT factors are also recruited to build protective subcellular environment against the RNAi machinery and other ribonucleases. Without the co-opted ESCRT factors, the tombusvirus VRCs seem to have permanent assembly deficiency, rendering the dsRNAs harbored within VRCs continuously exposed to the RNAi machinery, other host ribonucleases, and possibly cellular dsRNA sensors (Fig 8B).

The TBSV-induced spherules harboring VRCs are relatively stable membranous structures that likely synthesize viral progeny RNAs in cells for several hours, which occurs even *in vitro* when membranous VRCs are isolated [69]. However, the ESCRT proteins are known to act rapidly and temporarily in membrane budding events [70,71]. Therefore, TBSV likely involves additional cellular components to stabilize the vesicle-like structures in infected cells. Co-opted lipids are likely candidates for this function due to their known involvement in shaping membrane characteristics [72–74]. Accordingly, we have tested the role of sterols and phospholipids, which are critical for TBSV replication [43–47], in providing protection of viral RNA against RNAi. Indeed, deletion of ORPs or Scs2p VAP, which are known to affect MCS formation and co-opted by tombusviruses to enrich sterols at replication sites [45], resulted in reduced protection of the viral RNAs against RNAi in yeast or ribonucleases *in vitro*. Similarly, deletion of *ERG4* involved in ergosterol synthesis (the major cholesterol-like lipid in yeast) also sensitized TBSV against RNAi. Based on these data, we suggest that sterols likely facilitate the formation of more stable and durable spherules/VRCs. Similar to the co-opted ESCRT factors, enrichment of sterols in the replication compartment, and likely within individual spherules, seems to be required to assemble tombusvirus VRCs that are not continuously exposed to the RNAi machinery (Fig 8C). Interestingly, sterols are also thought to make the plasma membrane less permeable and wider [73].

In addition to sterols, we find PE and phospholipid levels also have a critical role in the formation of RNAi-insensitive replication compartment. Accordingly, the high PE level in *cho2Δ* yeast made the dsRNA replication intermediate less sensitive to RNAi in yeast and ribonuclease in vitro, suggesting more rapid and efficient VRC assembly when PE is abundant in membranes. The subverted PE molecules, due to their conical molecular structures, might facilitate the formation and stability of spherule structures by introducing negative curvature into lipid bilayers [73]. This model is further supported by an in vitro slicing assay, which demonstrated that purified FLAG-AGO1 from wt yeast showed slicing activity against TBSV RNA, whereas the purified FLAG-AGO1 from *cho2Δ* yeast showed negligible slicing activity, suggesting that DCR1 might have a limited access to TBSV dsRNA intermediates in *cho2Δ* yeast with high PE level.

Based on in vitro observations, we suggest that nucleases, including DCR1 and AGO1, can likely enter the VRC during the assembly process that makes the dsRNA sensitive to RNAi. Thus, rapid VRC assembly due to high PE level in *cho2Δ* yeast or high level of phospholipids in *opi1Δ* yeast decreases the time available for ribonucleases to associate with VRCs during their assembly process, thus leading to the enhanced protection of TBSV against RNAi in *cho2Δ* and *opi1Δ* yeasts. While in wt yeast the TBSV-induced local PE enrichment within the replication compartment takes up more time than the already PE-rich membranes in *cho2Δ* yeast. Thus the speed or efficiency of VRC assembly could be a key factor affecting the chance for the RNAi machinery to interact with viral RNAs harbored within VRCs.

The slower pace of VRC assembly process (Fig 8D) observed in wt versus *cho2Δ* or *opi1Δ* yeast CFEs (which could facilitate the entry of DCR1 and AGO1 into the forming VRCs) might also explain that continuous expression of DCR1 and AGO1 more efficiently inhibited TBSV repRNA accumulation in wt yeast than suppression of DCR1 and AGO1 expression during pre-growth (i.e., prior to induction of viral replication) in wt yeast (compare Figs 1 and 2A versus Figs 5 and 6A and S1A). The somewhat variable level of reduction in repRNA accumulation by the RNAi machinery in wt yeast background is likely due to differences among yeast colonies in their abilities to induce the expression of AGO1 and DCR1 from the pre-repressed *GAL1* promoter after the addition of galactose to the culture media.

The development of the reconstituted RNAi as a cellular probe also allowed us to demonstrate that the tombusvirus VRCs act as protective structures when assembled in the ER membranes (in the absence of peroxisomal membranes) similar to those assembled in the peroxisomal membranes. This observation indicates that TBSV could usurp the ER membrane and efficiently co-opt pro-viral host proteins and enrich lipids in this new subcellular location, giving high flexibility for this virus without sacrificing the protective quality and functionality of VRCs formed.

Overall, the reconstituted RNAi machinery of *S*. *castellii* in yeast, which supports the replication of TBSV RNA in membranous compartments, is useful intracellular probe to study the direct interaction between the RNAi machinery and the viral replicase complex, and the roles of subverted host factors in protecting the viral dsRNA replication intermediate from RNAi-based degradation. The interpretation of data in this system is simplified since *S*. *cerevisiae* does not code for an RNA-dependent RNA polymerase, which is important component of the RNAi machinery in plants and animals by producing dsRNA templates for amplifying the silencing signals [7,9]. Nevertheless, this work has demonstrated the role of co-opted cellular proteins and lipids in generation of membranous subcellular environment protected from RNAi to support TBSV replication. Other (+)RNA viruses also co-opt cellular proteins, including ESCRT factors, and subvert lipids for generation of membranous replication organelles [1,5,75–80], thus, it seems highly likely that our findings will also be applicable for wide-range of viruses.

**Summary:** By using the reconstituted RNAi in yeast, we have developed a simple intracellular probe to characterize membranous VRCs and viral replication compartments formed with the help of co-opted host factors in cells replicating TBSV. Moreover, we have compared the cellular data with in vitro replication results to gain deeper insights into the level of protection provided by the membranous VRCs against ribonucleases. These approaches have helped us uncover that the RNAi machinery and ribonucleases could harm viruses the most efficiently when one of two aspects of VRC assembly goes wrong. First, when the VRC assembly is permanently hindered by a missing co-opted host factor (such as the ESCRT proteins) or in the absence of local sterol enrichment in the replication compartment, then the dsRNA within the VRC is continuously exposed to RNAi or ribonucleases. Second, when the VRC assembly is slow due to a limiting host factor (such as PE accessibility), which possibly allows the components of RNAi machinery or ribonucleases to enter the VRCs prior to the completion of VRC assembly. This deficiency in excluding ribonucleases from VRCs due to slow assembly then leads to lower level of viral RNA accumulation.

## Materials and methods

### Yeast strains and expression plasmids

Yeast (*Saccharomyces cerevisiae)* strain BY4741 (*MAT***a** *his3*Δ*1 leu2*Δ*0 met15*Δ*0 ura3*Δ*0*) and the YKO library were obtained from Open Biosystems (Huntsville, AL, USA). Double deletion yeast strains ΔBro1::kanMX4,ΔVps23::hphNT1 and ΔVps20::kanMX4,ΔSnf7::hphNT1 were described previously [58]. SEY6210 (MATa *ura3-52 his3*Δ*200 lys2-801 leu2-3*, *trp1*Δ*901 suc2*Δ*9*), and JRY6266-his3 (SEY6210 *osh3*Δ::*LYS2 osh5*Δ::*LEU2 osh6*Δ::*LEU2 osh7*Δ::*kan-MX4*) were provided by Dr. Christopher T. Beh (Simon Fraser University) [81].

Yeast expression plasmids pESC-Ura-Gal10-HisDcr1 and pESC-Ura-Gal1-HisAgo1-Gal10-HisDcr1 were obtained as follows: First, *DCR1* and *AGO1* genes from *S*. *castellii* were PCR-amplified using primers #6015 (CCAGGAATTCATGGGTCATCATCATCATCATCATATGAATAGAGAAAAAAGCGCCGATC), #6016 (CCAGACTAGTTCACAGATTGTTGCAATGCCTC) and pRS315-Dcr1 [56] as a template for *DCR1* and primers #6013 (CCAGGTCGACATGGGTCATCATCATCATCATCATATGTCATCCAATTCGGAGGAG), #6014 (CCAGAAGCTTTCATATGTAGTACATGATGTCAGTG) and pRS314-Ago1 [56] as a template for *AGO1*. The obtained PCR product of *DCR1* was digested with EcoRI/SpeI and inserted into pESC-Ura plasmid (Stratagene), which was digested with EcoRI and SpeI to generate pESC-Ura-Gal10-HisDcr1. The PCR product of *AGO1* was digested with SalI/HindIII and inserted into pESC-Ura-Gal10-HisDcr1, which was digested with SalI and HindIII.

### Induction of RNAi through co-expression of DCR1 and AGO1 in yeast replicating TBSV repRNA

To study the effect of DCR1 and AGO1 co-expression on TBSV replication in yeast strains with ESCRT gene deletions, we co-transformed yeast strains BY4741 (parental, control), vps20Δ snf7Δ, vps20Δ and bro1Δvps23Δ with three plasmids: pGBK-HIS-Cup-Flag33-Gal-DI-72, pGAD-Cup-Flag92 and one of the following: pESC-Ura (used as a control), pESC-Ura-Gal10-HisDcr1 or pESC-Ura-Gal1-HisAgo1-Gal10-HisDcr1. Transformed yeast cells were selected on SC-ULH^-^ plates and pre-grown in 1 ml SC-ULH^-^ media supplemented with 2% glucose and 100 μM BCS for 24 h at 29°C. Yeast cells were then centrifuged at 2,000 rpm for 3 min, washed with SC-ULH^-^ media supplemented with 2% galactose and resuspended in 2 ml SC-ULH^-^ media with 2% galactose and 100 μM BCS followed by culturing at 29°C for 24 h to express AGO1 and DCR1 prior to initiation of replication. Then, yeast cells were centrifuged at 2,000 rpm for 3 min, washed with SC-ULH^-^ media supplemented with 2% galactose and resuspended in 3 ml SC-ULH^-^ media with 2% galactose and 50 μM CuSO_4_, followed by culturing yeast cells at 23°C for 16 h (Fig 2) or 24 h (Fig 1), and then processed for total RNA and protein extractions. Northern blotting and Western blotting were done as previously published [53].

Yeast strains BY4741, ino2Δ, scs2Δ, and erg4Δ were transformed with plasmids pGAD-CUP1-His-p92 (Leu2 selection), pGBK-CUP1-His-p33-ADH1-DI72 (His3 selection), and pESC-Ura-Gal1-HisAgo1-Gal10-HisDcr1 (Ura3 selection), pESC-Ura-Gal10-HisDcr1 or pESC empty. Transformed yeast were pre-grown at 23°C for 24 h in SC-ULH^-^ media supplemented with 2% glucose and 100 μM BCS. After cell harvest and a washing step, the yeasts were grown at 23°C for 16 h (for 8 h in case of pex3Δ yeast), in SC-ULH^-^ media supplemented with 2% galactose and 100 μM BCS. Then, yeast cells were centrifuged at 2,000 rpm for 3 min, washed with SC-ULH^-^ media supplemented with 2% galactose and resuspended in SC-ULH^-^ media with 2% galactose and 50 μM CuSO_4_, followed by culturing yeast cells at 23°C for 16 or 24 h, followed by RNA and protein analysis as described [53].

BY4741, cho2Δ and opi1Δ yeasts were transformed with plasmids pGAD-CUP1-His-p92 (Leu2 selection), pGBK-CUP1-His-p33-Gal1-DI72 (His3 selection), and pESC-Ura-Gal1-HisAgo1-Gal10-HisDcr1 (Ura3 selection), pESC-Ura-Gal10-HisDcr1 or pESC-empty. Transformed yeasts were pre-grown at 23°C for 24 h in SC-ULH^-^ media supplemented with 2% galactose and 100 μM BCS. Then, yeast cells were centrifuged at 2,000 rpm for 3 min, washed with SC-ULH^-^ media supplemented with 2% galactose and resuspended in SC-ULH^-^ media with 2% galactose and 50 μM CuSO_4_, followed by culturing yeast cells at 23°C for 16 h, followed by RNA and protein analysis as described [53].

SEY6210 and JRY6266-his3 yeast strains were transformed with plasmids pGAD-CUP1-His-p92 (Trp1 selection), pGBK-CUP1-His-p33-Gal1-DI72 (His3 selection), and pESC-Ura-Gal1-HisAgo1-Gal10-HisDcr1 (Ura3 selection), pESC-Ura-Gal10-HisDcr1 or pESC-empty. Transformed yeasts were pre-grown at 29°C overnight in SC-UTH^-^ media supplemented with 2% galactose and 100 μM BCS. After cell harvest and a washing step, the yeasts were grown at 29°C for 16 h in SC-UTH^-^ media with 2% galactose and 50 μM CuSO_4_, followed by RNA and protein analysis as described [53].

### In vitro TBSV replication assay #1

To support *in vitro* TBSV replication, cell-free extracts (CFE) were prepared from untransformed BY4741, snf7Δ, vps20Δ, snf7Δvps20Δ, bro1Δ, vps23Δ and bro1Δvps23Δ yeast strains as described earlier [42], whereas CFEs were obtained from cho2Δ, opi1Δ, and BY4741 (control) as described in [43]. Reaction mixture for the *in vitro* TBSV replication contained 2 μl of CFE, 0.15 μg (+) DI-72 RNA, 400 ng affinity-purified MBP-p33, 400 ng affinity-purified MBP-p92^pol^ in 20 μl total volume. The reactions were performed for 3 h at 25°C.

### In vitro TBSV replication assay #2

To support *in vitro* TBSV replication, CFEs were prepared from BY4741, ino2Δ, scs2Δ, erg4Δ, pex3Δ, SEY6210 and JRY6266 yeast strains, which were transformed with pESC-CUP1-p92 (Ura3 selection) and pGBK-CUP1-p33 (His3 selection). The CFE-based reaction mixtures were programmed with 0.5 μg DI-72(+) RNA transcripts as described [42,69]. The CFE-based replication mixtures were incubated at 25°C for 3 h.

### Ribonuclease treatment of the in vitro TBSV replication products

Treatments of the RNA products from the CFE-based TBSV replication reactions (for Fig 1B) with RNases were done as follows: After 1 h incubation at 25°C, the TBSV replication products were treated with both ssRNA-specific RNase A (VWR) and dsRNA-specific ribonuclease RNase III (NEB). After incubation at 37°C for either 15 or 20 min, the RNA samples were extracted with phenol-chloroform and precipitated.

Treatments of *in vitro* assembled VRCs in CFEs with the micrococcal nuclease (Amersham) were performed as follows: MNase (final concentration of 0.1 or 0.05 U/μl in the presence of 1 mM CaCl_2_) was added at different time points to CFE mixtures as shown in Figures. The reaction mixtures were incubated for 15 or 20 min at room temperature and, then, 2.5 mM EGTA was added to the samples to inactivate the MNase. The CFE reactions were further incubated for a total of 3 h (counted from the start of the replication assay) at 25°C before the products were extracted with phenol-chloroform and precipitated. The obtained ^32^P-labeled RNA products were separated by electrophoresis in 5% semi-denaturing polyacrylamide gel containing 8 M urea with 0.5x Tris-borate-EDTA buffer [50,58].

The CFE-based TBSV replication assay #2 was performed in the presence of 0.5 U RNase III (NEB) during the entire incubation (3 hours at 25°C). Then, the RNA samples were extracted with phenol-chloroform and precipitated. The obtained ^32^P-labeled RNA products without heat treatment were analyzed in 5% acrylamide/ 8M Urea gels to detect dsRNA level [50].

## Supporting information

S1 Fig (A) Separate expression of AGO1 and DCR1 does not affect TBSV replication in yeast (*Saccharomyces cerevisiae*) with reconstituted RNAi machinery. *S*. *castellii AGO1* and *DCR1* genes under the control of yeast *TEF1* constitutive promoter were integrated into *S*. *cerevisiae* chromosomes to generate yeast RNAi strains expressing AGO1 or DCR1, or co-expressing both AGO1 and DCR1. TBSV repRNA replication was induced in RNAi yeast strains and total RNA samples were analyzed in a denaturing PAGE gel (stained with Ethidium-bromide) to detect the viral repRNA accumulation. 5S ribosomal RNA is used as a loading control. Note that the AGO1 samples are underloaded. Co-expression of AGO1 and DCR1 from the strong constitutive *TEF1* promoter reduced TBSV repRNA accumulation below detection level. (B) Inducible RNAi yeast strains were generated by integration of *AGO1* and *DCR1* genes controlled by the galactose-inducible promoter *GALL* (a version of *GAL1*) into yeast chromosomes. Plasmids required for copper-inducible expression of TBSV p33, p92, and DI-72 repRNA were transformed into these yeast strains. Yeast cells were grown in galactose-containing media to induce the expression of RNAi components. Virus replication was induced by addition of CuSO_4_ into growth media, and total RNA samples were isolated 24 hours after induction. TBSV repRNA accumulation and 18S ribosomal RNA level was tested by Northern blotting from total RNA samples. (C) Northern blot detection of vsiRNAs from yeast samples shown in panel B. Total RNA samples were hybridized to ^32^P-labeled oligonucleotides annealed to different regions of TBSV RNA. The sizes of the 22- and 24-nt RNA markers are indicated next to lane 4.
(TIF)

S2 Fig Reduced ergosterol enrichment in the viral replication compartment increases the sensitivity of replicating tombusvirus RNA to the reconstituted RNAi in yeast. (A-B) Induction of RNAi in *scs2Δ* yeast inhibits TBSV repRNA accumulation. Top panels: Replication of the TBSV repRNA was measured by Northern blotting 24 h after initiation of TBSV replication. See Fig 1A for further details. (C) Non-denaturing PAGE analysis of the ^32^P-labeled TBSV repRNA products obtained in the CFE-based assay. The CFEs were prepared from BY4741 or *scs2Δ* yeast strains, which expressed p33 and p92, and programmed with (+)repRNA. Samples were treated with the dsRNA-specific RNase III during the entire assay to target accessible dsRNA replication intermediates. See further details in the legends for Fig 3.
(TIF)

S3 Fig Reduced phospholipid biosynthesis in yeast enhances the sensitivity of replicating tombusvirus RNA to the reconstituted RNAi. (A-B) Induction of RNAi in *ino2Δ* yeast inhibits TBSV repRNA accumulation more effectively than in wt yeast. Top panels: Replication of the TBSV repRNA was measured by Northern blotting 24 h after initiation of TBSV replication. See Fig 1A for further details. (C) Non-denaturing PAGE analysis of the ^32^P-labeled TBSV repRNA products obtained in the CFE-based assay. The CFEs were prepared from BY4741 or *ino2Δ* yeast strains, which expressed p33 and p92, and programmed with (+)repRNA. Samples were treated with the dsRNA-specific RNase III during the entire assay to target accessible dsRNA replication intermediates. See further details in the legends for Fig 3.
(TIF)

S1 Text Small interfering RNA (vsiRNA) detection from yeast cells. (DOC)
