# Supplementary figures and images for: The role of co-opted ESCRT proteins and lipid factors in protection of tombusviral double-stranded RNA replication intermediate against reconstituted RNAi in yeast
Source: PLoS Pathog. 2017 Jul 31;13(7):e1006520. doi: 10.1371/journal.ppat.1006520 (PMC5552349; doi:10.1371/journal.ppat.1006520)

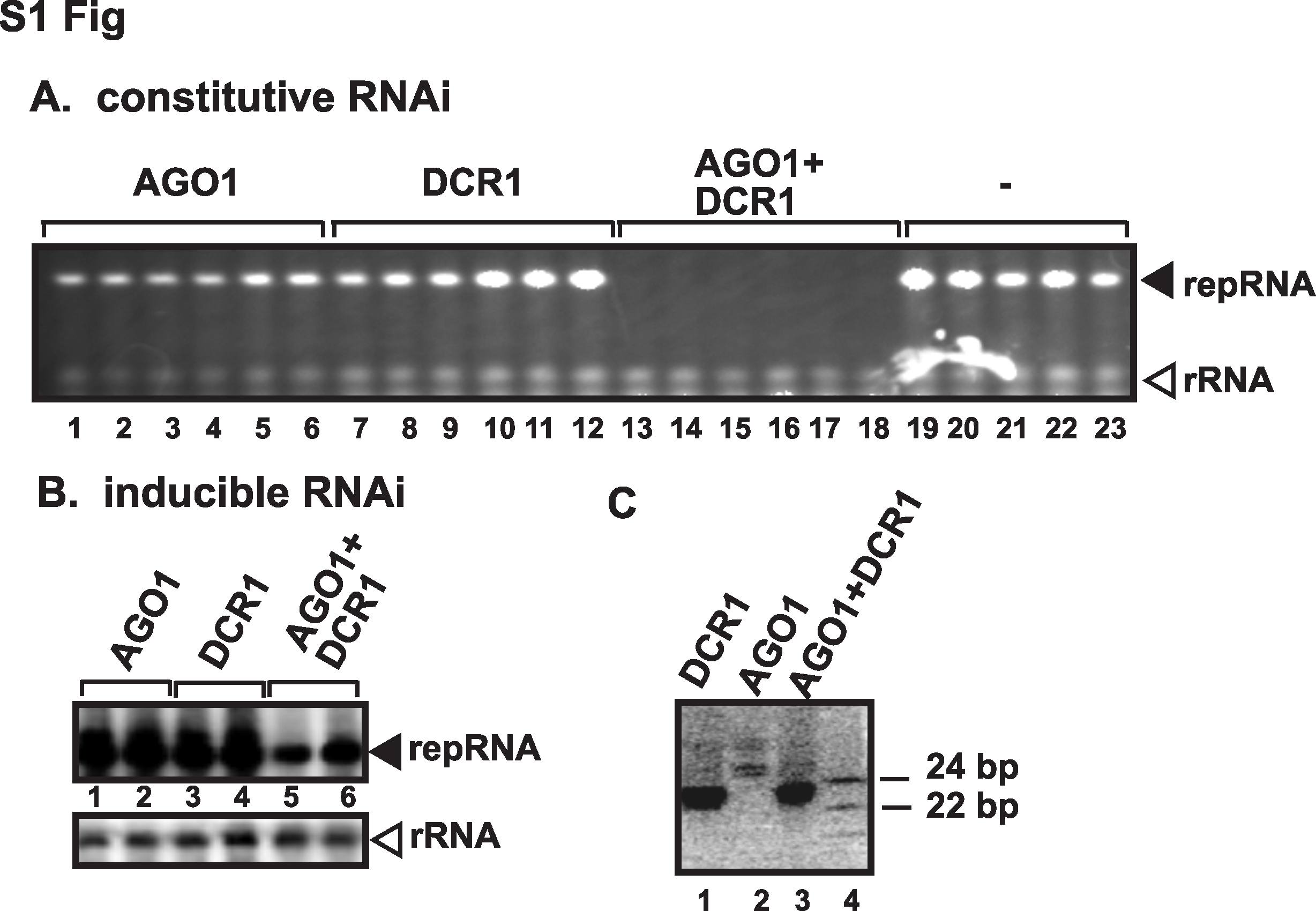

Supplement: S1 Fig — (A) Separate expression of AGO1 and DCR1 does not affect TBSV replication in yeast (Saccharomyces cerevisiae) with reconstituted RNAi machinery. S. castellii AGO1 and DCR1 genes under the control of yeast TEF1 constitutive promoter were integrated into S. cerevisiae chromosomes to generate yeast RNAi strains expressing AGO1 or DCR1, or co-expressing both AGO1 and DCR1. TBSV repRNA replication was induced in RNAi yeast strains and total RNA samples were analyzed in a denaturing PAGE gel (stained with Ethidium-bromide) to detect the viral repRNA accumulation. 5S ribosomal RNA is used as a loading control. Note that the AGO1 samples are underloaded. Co-expression of AGO1 and DCR1 from the strong constitutive TEF1 promoter reduced TBSV repRNA accumulation below detection level. (B) Inducible RNAi yeast strains were generated by integration of AGO1 and DCR1 genes controlled by the galactose-inducible promoter GALL (a version of GAL1) into yeast chromosomes. Plasmids required for copper-inducible expression of TBSV p33, p92, and DI-72 repRNA were transformed into these yeast strains. Yeast cells were grown in galactose-containing media to induce the expression of RNAi components. Virus replication was induced by addition of CuSO4 into growth media, and total RNA samples were isolated 24 hours after induction. TBSV repRNA accumulation and 18S ribosomal RNA level was tested by Northern blotting from total RNA samples. (C) Northern blot detection of vsiRNAs from yeast samples shown in panel B. Total RNA samples were hybridized to 32P-labeled oligonucleotides annealed to different regions of TBSV RNA. The sizes of the 22- and 24-nt RNA markers are indicated next to lane 4. (TIF) [file ppat.1006520.s001.tif]

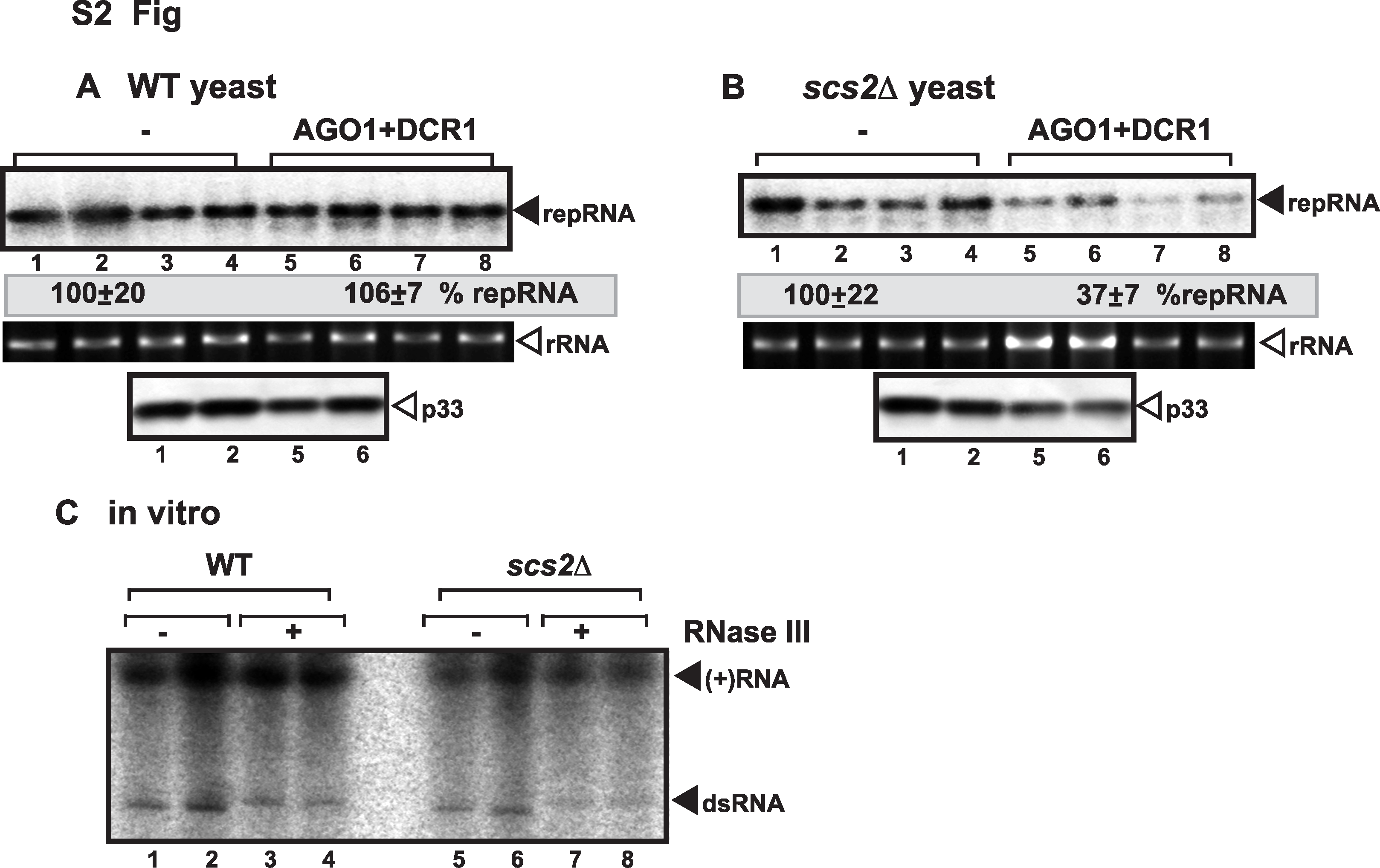

Supplement: S2 Fig — (A-B) Induction of RNAi in scs2Δ yeast inhibits TBSV repRNA accumulation. Top panels: Replication of the TBSV repRNA was measured by Northern blotting 24 h after initiation of TBSV replication. See Fig 1A for further details. (C) Non-denaturing PAGE analysis of the 32P-labeled TBSV repRNA products obtained in the CFE-based assay. The CFEs were prepared from BY4741 or scs2Δ yeast strains, which expressed p33 and p92, and programmed with (+)repRNA. Samples were treated with the dsRNA-specific RNase III during the entire assay to target accessible dsRNA replication intermediates. See further details in the legends for Fig 3. (TIF) [file ppat.1006520.s002.tif]

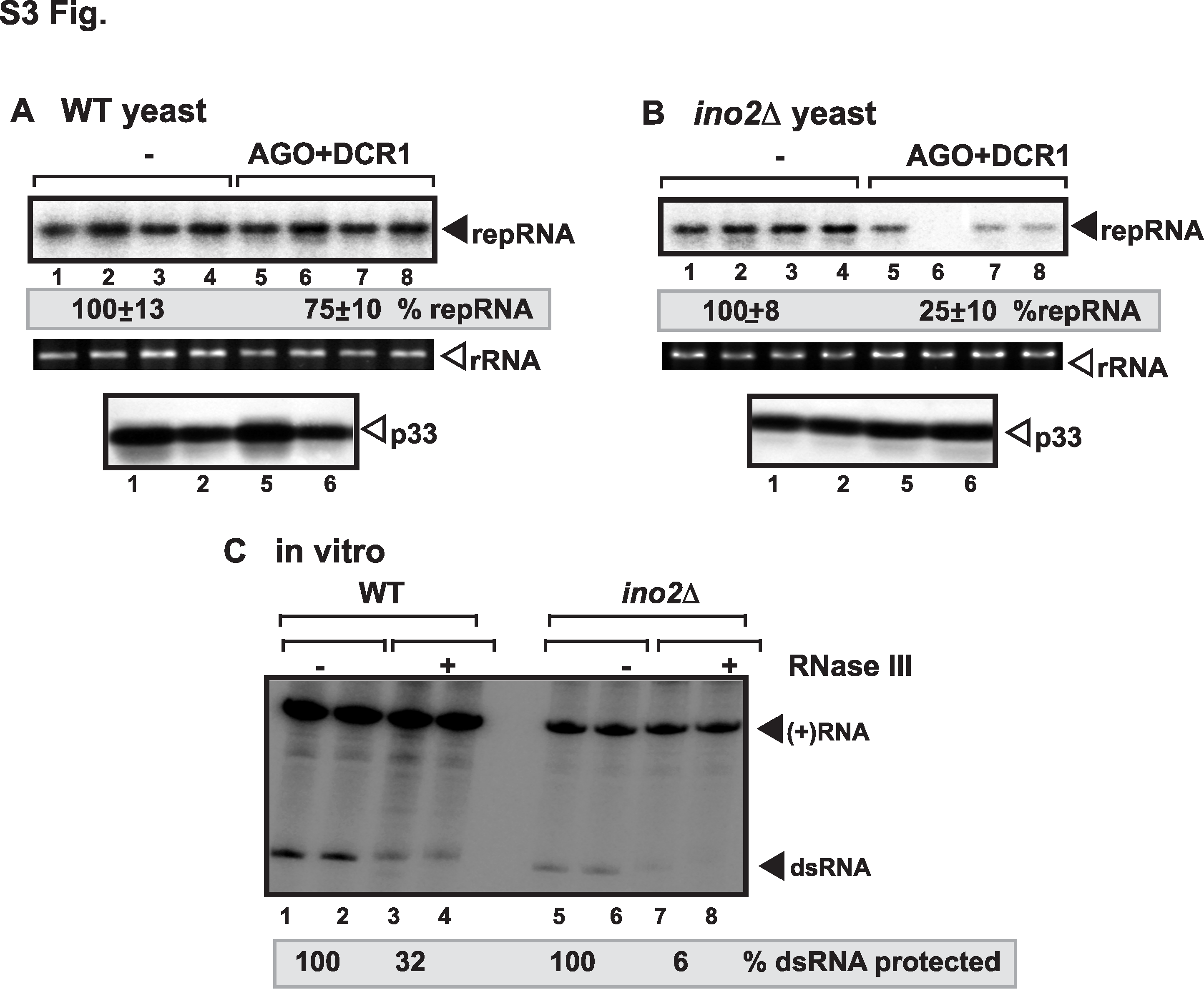

Supplement: S3 Fig — (A-B) Induction of RNAi in ino2Δ yeast inhibits TBSV repRNA accumulation more effectively than in wt yeast. Top panels: Replication of the TBSV repRNA was measured by Northern blotting 24 h after initiation of TBSV replication. See Fig 1A for further details. (C) Non-denaturing PAGE analysis of the 32P-labeled TBSV repRNA products obtained in the CFE-based assay. The CFEs were prepared from BY4741 or ino2Δ yeast strains, which expressed p33 and p92, and programmed with (+)repRNA. Samples were treated with the dsRNA-specific RNase III during the entire assay to target accessible dsRNA replication intermediates. See further details in the legends for Fig 3. (TIF) [file ppat.1006520.s003.tif]
