# Supplementary material for: The role of co-opted ESCRT proteins and lipid factors in protection of tombusviral double-stranded RNA replication intermediate against reconstituted RNAi in yeast
Source: PLoS Pathog. 2017 Jul 31;13(7):e1006520. doi: 10.1371/journal.ppat.1006520 (PMC5552349; doi:10.1371/journal.ppat.1006520)
Supplement: S1 Text — (DOC) [file ppat.1006520.s004.doc]

**SUPPLEMENT:**

**Supplementary Materials and Methods:**

**Small interfering RNA (vsiRNA) detection from yeast cells.** Yeast strains erg4△, vps23△bro1△, and BY4741 replicating TBSV repRNA and expressing DCR1, AGO1, or DCR1/AGO1 were used to obtain total RNA based on the standard hot phenol method [1]. RNA pellet was dissolved in water with addition of equal amounts of PEG solution (20% PEG 8000, 2 M NaCl). Samples were mixed and incubated for 1 h on ice, followed by centrifugation at 14,000g for 15 min. vsiRNAs were precipitated from the supernatant by ethanol and dissolved in water and added equal amount 2x Urea-TE loading buffer (8 M Urea, 10 mM Tris-HCl pH 8.0, 50 mM EDTA, 0.05% w/v bromphenol blue, 0.05% w/v xylene cyanol FF) and heated at 85°C for 5 min. The vsiRNAs were separated on a 15% polyacrylamide/8 M urea gel, followed by transfer onto Hybond-N+ membrane (Roche). Crosslinking was performed by 1-ethyl-3-(3-dimethylaminopropyl) (EDC) [2]. Whatman paper (membrane size) was saturated with EDC solution. Membrane with the RNA side on top was placed on the EDC-saturated Whatman paper, wrapped in cling film, and incubated for 2 h at 60°C. Crosslinked membranes were pretreated with ULTRAhyb-Oligo Hybridization buffer (Ambion) for at 1 h at 40°C, followed by the addition of ^32^P-labeled DI-72 (-)RNA probe overnight at 40°C. The membrane was washed by 2x SSC with 0.1% SDS two times for 15 min at 40 °C. The bound probes were detected using Phosphorimager.

**Purification of FLAG-AGO1 protein from yeast.** Yeast strain BY4741 was transformed with plasmids pRS315-pGall-HADcr1, pRS316-pGall-FlagAgo1/pCup-Hisp92 and either pGBK-HIS-Cup-Flag33/Gal-DI-72 [3] to launch replication of TBSV DI-72 repRNA or pESC(His)-pCup-FHV-RNA1 [4] to launch replication of FHV RNA1. The transformed yeast cells were selected on SC-ULH^−^ plates and pre-grown in SC-ULH^−^ media supplemented with 2% glucose and 100 μM BCS at 23°C. Yeast cells were centrifuged at 2,000 rpm for 3 min and then, washed with SC-ULH^−^ media supplemented with 2% galactose. Yeast cells were resuspended in SC-ULH^−^ media supplemented with 2% galactose and 50 µM CuSO_4_. After growing for 24 h at 23°C, yeast cells were pelleted and homogenized by glass beads using FastPrep Homogenizer (MP Biomedicals) in HS-TG buffer [50 mM Tris–HCl (pH 7.5), 10% glycerol, 15 mM MgCl_2_, 10 mM KCl, 0.5 M NaCl, and 1% [V/V] yeast protease inhibitor cocktail (Ypic)]. Membrane fraction was separated by centrifugation for 20 min at 42,000 g. The supernatant (soluble fraction) was incubated with anti-FLAG M2 resin at 4°C for 3 hours. After washing columns 3 times with LS-TG buffer [50 mM Tris–HCl [pH 7.5], 10% glycerol, 15 mM MgCl_2_, 10 mM KCl, 50 mM NaCl], FLAG-AGO1 protein was eluted in 80 µl of LS-TG buffer supplemented with 0.15 mg/ml Flag peptide (Sigma). Purified FLAG-AGO1 protein was detected by Western blot using anti-Flag antibody. Primary detection was followed by anti-mouse antibody conjugated to alkaline phosphatase. Colorimetric detection was performed with NBT and BCIP.

**In vitro slicing assay with purified FLAG-AGO1 protein.** The ^32^P-labeled TBSV gRNA transcripts were generated using T7 transcription as described [5]. Briefly, PCR reactions were performed with pT100 as template and primers #359 (GTAATACGACTCACTATAGGAAATTCTCCAGGATTTC) and #1190 (GGGCTGCATTTCTGCAATG). T7 transcription reactions were performed and obtained transcripts were purified as described earlier [5].

In vitro cleavage (slicing) reactions were carried out for 1 h at 25 °C in the RdRp buffer (50 mM Tris-HCl (pH 8.2), 10 mM MgCl_2_, 10 mM dithiothreitol), containing 6 U of RNasin, 0.1 pmol of ^32^P-labeled RNA probes, 0.1 μg tRNA and 1 μl of affinity-purified (pre-loaded with vsiRNA) FLAG-AGO1 in a 10 μl reaction volume. The RNA samples were extracted with phenol-chloroform, precipitated and separated by electrophoresis in a denaturing 5% polyacrylamide gel containing 8 M urea with 0.5x Tris-borate-EDTA buffer.

**REFERENCES:**

1. Li Z, Pogany J, Panavas T, Xu K, Esposito AM, et al. (2009) Translation elongation factor 1A is a component of the tombusvirus replicase complex and affects the stability of the p33 replication co-factor. Virology 385: 245-260.

2. Pall GS, Hamilton AJ (2008) Improved northern blot method for enhanced detection of small RNA. Nat Protoc 3: 1077-1084.

3. Kovalev N, Nagy PD (2013) Cyclophilin a binds to the viral RNA and replication proteins, resulting in inhibition of tombusviral replicase assembly. J Virol 87: 13330-13342.

4. Kovalev N, Pogany J, Nagy PD (2012) A Co-Opted DEAD-Box RNA Helicase Enhances Tombusvirus Plus-Strand Synthesis. PLoS Pathog 8: e1002537.

5. Rajendran KS, Nagy PD (2003) Characterization of the RNA-binding domains in the replicase proteins of tomato bushy stunt virus. J Virol 77: 9244-9258.
